# An energy budget agent-based model of earthworm populations and its application to study the effects of pesticides

**DOI:** 10.1016/j.ecolmodel.2013.09.012

**Published:** 2014-05-24

**Authors:** A.S.A. Johnston, M.E. Hodson, P. Thorbek, T. Alvarez, R.M. Sibly

**Affiliations:** a School of Biological Sciences, University of Reading, UK; b Environment Department, University of York, UK; c Environmental Safety, Syngenta Ltd., Bracknell, UK; d EcoRisk Solutions Ltd., Norwich, UK

**Keywords:** Energy budget model, Earthworm, Agent based model, Toxic stress, Mechanistic effect model, Pesticides

## Abstract

Earthworms are important organisms in soil communities and so are used as model organisms in environmental risk assessments of chemicals. However current risk assessments of soil invertebrates are based on short-term laboratory studies, of limited ecological relevance, supplemented if necessary by site-specific field trials, which sometimes are challenging to apply across the whole agricultural landscape. Here, we investigate whether population responses to environmental stressors and pesticide exposure can be accurately predicted by combining energy budget and agent-based models (ABMs), based on knowledge of how individuals respond to their local circumstances. A simple energy budget model was implemented within each earthworm *Eisenia fetida* in the ABM, based on a priori parameter estimates. From broadly accepted physiological principles, simple algorithms specify how energy acquisition and expenditure drive life cycle processes. Each individual allocates energy between maintenance, growth and/or reproduction under varying conditions of food density, soil temperature and soil moisture. When simulating published experiments, good model fits were obtained to experimental data on individual growth, reproduction and starvation. Using the energy budget model as a platform we developed methods to identify which of the physiological parameters in the energy budget model (rates of ingestion, maintenance, growth or reproduction) are primarily affected by pesticide applications, producing four hypotheses about how toxicity acts. We tested these hypotheses by comparing model outputs with published toxicity data on the effects of copper oxychloride and chlorpyrifos on *E. fetida*. Both growth and reproduction were directly affected in experiments in which sufficient food was provided, whilst maintenance was targeted under food limitation. Although we only incorporate toxic effects at the individual level we show how ABMs can readily extrapolate to larger scales by providing good model fits to field population data. The ability of the presented model to fit the available field and laboratory data for *E. fetida* demonstrates the promise of the agent-based approach in ecology, by showing how biological knowledge can be used to make ecological inferences. Further work is required to extend the approach to populations of more ecologically relevant species studied at the field scale. Such a model could help extrapolate from laboratory to field conditions and from one set of field conditions to another or from species to species.

## Highlights

• We model *Eisenia fetida* populations using an agent-based framework.
• Individual energy budgets follow the basic principles of physiological ecology.
• Methods are developed to show how chemicals achieve their physiological effects.
• The model realistically captures sublethal effects under variable feeding conditions.
• Energy budget ABMs have potential for refining chemical risk assessment.

## 1 Introduction

Earthworms are significant contributors to the ecosystem services provided by arable soils (Daily et al., 1997; Keith and Robinson, 2012; Blouin et al., 2013) and they respond rapidly to alterations in soil quality (Fraser et al., 1996), tillage (Chan, 2001) and exposure to chemical toxicants (Vorphal et al., 2009). Consequently, they are focal organisms for environmental risk assessments of agricultural chemicals in Europe (OECD, 1984). However, current regulatory guidance for risk assessment is limited to short-term laboratory studies supplemented if necessary by site-specific field trials to investigate population-level effects (SANCO, 2002). Laboratory studies have limited ecological relevance since they are carried out in standardised conditions, whilst field trials are expensive, time-consuming and challenging to interpret for a wide range of agricultural landscapes (Jänsch et al., 2006). Here, we investigate a mechanistic approach to modelling organism responses to environmental and chemical exposure. This approach has the potential to act as a refinement option for chemical risk assessments if it can accurately predict population-level responses to the agricultural uses of plant protection products (PPPs) under a range of different conditions (Thorbek et al., 2010).

As individual physiologies direct the life cycle processes (e.g. growth and reproduction) which give rise to a population's dynamics, modelling at the individual level is crucial in mechanistic effects modelling for chemical risk assessment (Grimm et al., 2005; DeAngelis and Mooij, 2005). Individual physiology can be described by energy budgets and modelled using well-established principles of energy and mass conservation (Sousa et al., 2010; Sibly et al., 2013). Organisms uptake resources (in the form of food) from their environment and expend assimilated energy on maintenance, growth and reproduction (Karasov and Martinez del Rio, 2007; Sibly and Calow, 1986), but the allocation of energy to metabolic processes depends on a combination of environment- and organism-specific conditions (e.g., Nisbet et al., 2000). Dynamic energy budget (DEB) theory (Kooijman, 2010) provides a method for modelling individual physiology and DEB models have been previously developed for earthworm species by Baveco and de Roos (1996) and Klok et al. (2007). We do not follow DEB theory here because DEB models implement a ‘kappa rule’ which assumes that a fixed proportion of assimilated resources are allocated to maintenance and growth and the remainder to reproduction throughout life. It is important to realise that the kappa rule is (1) not in accord with the principles of physiological ecology as outlined in e.g. Karasov and Martinez del Rio (2007) and Sibly and Calow (1986); (2) denies the possibility of allocation trade-offs between growth and reproduction which are widely believed to occur (see e.g. Stearns, 1992); (3) has been shown not to apply to *Daphnia magna*, the species for which DEB was initially devised, by Glazier and Calow (1992) and Nisbet et al. (2004), who showed the proportion allocated to growth reduces from 1 early in life to <0.05 later; (4) is contradicted by the finding that in the absence of a sexual partner, earthworms grow larger, indicating that reproduction has priority over growth in adults (Neuhauser et al., 1980) and; (5) is contradicted by observations of continued reproduction during weight loss by earthworms under limiting feeding conditions (Reinecke and Viljoen, 1990). Baveco and de Roos (1996) and Klok et al. (2007) offer no evidence that the kappa rule applies to earthworms. We therefore felt it necessary to develop a more accurate mechanistic energy budget model based on accepted principles of physiological ecology and have followed the approach of Sibly et al. (2013).

The purpose of this study is to develop and evaluate a mechanistic model of earthworm responses to local conditions, describing physiological responses to biotic and abiotic factors at the individual level, and seeing how this translates to the population level. We construct an energy-budget-driven ABM for the earthworm species *Eisenia fetida*, and compare model outputs to experimental data from the literature on both individual life cycle processes and population dynamics. In the experiments we simulated, individuals were kept for periods of time with depleting food supplies. To simulate these experiments mechanistically, model landscapes incorporating spatially and temporally varying food availability are required, so that the necessary interactions between individuals, stress exposure and lack of food can occur. Following its success in simulating published data from non-toxic environments, we develop and evaluate methods for considering how individual physiological processes are altered by toxic stress. Adopting the methodology of Jager and Zimmer (2012) we assume that pesticides impose stress on specific physiological parameters, which have predictable effects on growth, reproduction and/or starvation following energy allocation principles. Although not common in the field, *E. fetida* is used as a model species due to the ample quantity of literature data available for model development and evaluation at the individual level. However, we anticipate that our model can be developed for application to other species and environmental conditions.

## 2 Methods

Here we provide a full model description and give an outline summary of model evaluation methods. Full details following guidelines for transparent and comprehensive ecological modelling documentation (TRACE) (Schmolke et al., 2010) are presented in the supplementary material.

### 2.1 Model description

The model description follows the ODD (Overview, Design concepts, and Details) protocol for describing ABMs (Grimm et al., 2010). The model is implemented in Netlogo 5.0.2 (Wilensky, 1999), a platform for building ABMs. The Netlogo code is available in supplementary material.

#### 2.1.1 Purpose

The purpose of the model is to simulate *Eisenia fetida* population dynamics under varying environmental conditions representative of those encountered in the field and investigate how energy budgets can be used to investigate how pesticides achieve their physiological effects.

#### 2.1.2 Entities, state variables and scales

This ABM comprises a number of individual *E. fetida* individuals and a model landscape consisting of two-dimensional 0.01 m^2^ patches of soil. Individuals are characterised by life cycle stage (cocoon, juvenile or adult), mass and energy reserves, and landscape patches by food density, soil temperature, soil moisture and pesticide concentration. The model proceeds in discrete daily time-steps. Metabolic calculations are in units of energy per unit time (kJ/day).

#### 2.1.3 Process overview and scheduling

**Fig. 1:**
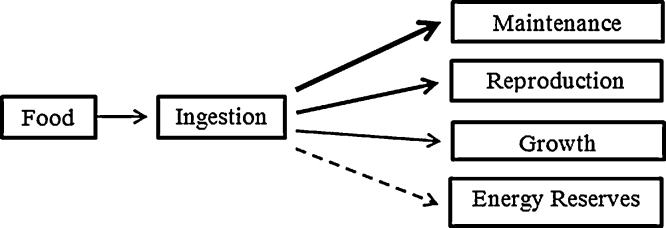
Structure of the energy budget model for adult *E. fetida*, with the thickness of arrows indicating priorities for energy allocation from food. Cocoons and juveniles are also in the model though cocoons do not grow and juveniles do not reproduce. Energy remaining after allocation enters the energy reserves which may be used for other functions when food is limited.

Each individual in the ABM has its own energy budget. The energy budget model includes algorithms for how energy uptake and expenditure direct life cycle processes based on fundamental principles of physiological ecology, and generally follows the methodology of Sibly et al. (2013). Individuals assimilate energy from ingested food (*Ingestion and Energy Uptake*) and expend available energy on maintenance (*Maintenance*), growth (*Growth*) and reproduction (*Reproduction*) in the order of priority outlined in Fig. 1. Total available energy is limited by the amount of food an organism can ingest, whilst mass and temperature have scaling effects on individual metabolic rates (Brown et al., 2004). Maintenance is essential for the survival of an individual, and thus has first priority for energy allocation. Juveniles grow until sexually mature, and thereafter adults preferentially allocate energy to reproduction before growth. If energy remains after reproduction and/or growth, energy is stored in the energy reserves as glycogen (Byzova, 1977), which may be used to pay maintenance costs when food is limited (*Energy Reserves and Starvation*).

**Fig. 2:**
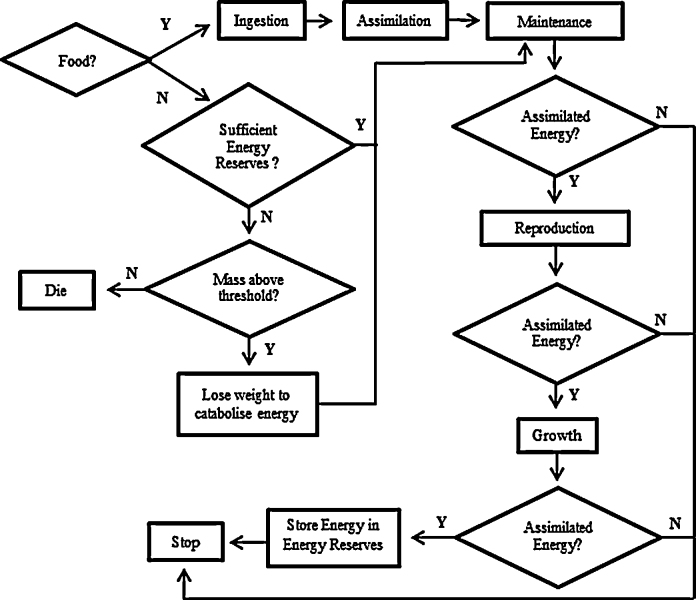
Partial energy flow diagram of *E. fetida* adults within the ABM, showing the processes (rectangles) each individual goes through per time step, with diamonds indicating decision points. Energy reserves are used for maintenance and reproduction in starving individuals.

Juveniles and adults move randomly in the landscape (*Movement*), assimilating a fixed proportion of energy from ingested food that fuels life cycle processes and survival. Feeding by individuals depletes landscape patches and the food density changes accordingly. Cocoons cannot feed or move but pay maintenance costs from energy reserves until they are fully developed at the end of the temperature-dependent incubation period, when they hatch as juveniles (Sousa et al., 2010). Juveniles transform to adults once they reach a body mass threshold for sexual maturity (Ma, 1984; Springett and Gray, 1992). Food was provided in the same amounts as in the experiment being simulated, and food densities in landscape patches depleted as individuals ingested food. When food was not available, energy reserves were used to cover maintenance costs. Once the energy reserves are depleted to a critical level individuals catabolise energy from tissue to meet maintenance demands (*Survival*). Pesticides were applied in the ABM at the concentrations and times specified in the experiment being simulated. Individuals experiencing these concentrations were affected as indicated by potential ‘toxicity submodels’. Fig. 2 gives an overview of processes occurring at the adult stage in each time-step under different feeding conditions.

#### 2.1.4 Design concepts

*Basic principles.* Key processes in the model determine how energy consumption and expenditure direct life cycle processes in response to environmental and pesticide exposure. Individual energy budgets follow fundamental principles of physiological ecology (Sibly and Calow, 1986) and scale with body mass and temperature according to known allometric laws (Sibly et al., 2013). Pesticides achieve their effects by imposing stress on specific physiological parameters following a dose-response relationship obtained from toxicity data.

*Emergence.* Variation in food availability between patches arises from the random movement and feeding of individuals in the landscape. Population dynamics emerge from differential energy allocation amongst individuals which is affected by food availability, soil temperature, soil moisture and pesticide concentration (Reinecke and Viljoen, 1990; Tripathi and Bhardwaj, 2004; Edwards and Bater, 1992).

*Interaction.* Individuals need mates (any other adult as earthworms are hermaphrodite (Dominguez et al., 2003)) present in the same patch to reproduce. Adults and juveniles interact indirectly by competing for food within patches, and both affect patches by depleting food.

*Stochasticity.* Movement and background mortality are random amongst juveniles and adults, with specified probability density functions.

*Observation*. Population density, stage class structure (cocoon, juvenile, adult) and individual body masses and reproduction were recorded.

#### 2.1.5 Initialisation

Simulations were initialised with individuals randomly distributed in the landscape. Landscape size and earthworm numbers, life cycle stages and body masses followed the experiments being replicated, outlined in detail in Section 2.2.

#### 2.1.6 Input data

The model does not utilise any input data for representing external driving factors.

#### 2.1.7 Submodels

Species-specific parameters were derived from the literature for *E. fetida* as shown in Table 1. Where data were not available for *E. fetida* closely related species were used. For example, the assimilation efficiency estimated by Hobbelen and van Gestel (2007) was for *Lumbricus rubellus*, which we suggest is similar for *E. fetida* given its epigeic feeding strategy and additional support provided in Appendix A. A number of assumptions about the metabolism of individuals were necessary for model development and these are described in the following sections. Further details of parameter calculations are available in supplementary material (Appendix A). The following sections describe the energy budget model, outlined in the above sections and in Fig. 1, in terms of metabolic organisation at the individual level.

##### 2.1.7.1 Maintenance

The basal metabolic rate (*B*) is the level of metabolism below which an organism cannot survive (Fry, 1971; Calow and Sibly, 1990), and is used here as a measure of maintenance costs. Costs of movement, small in earthworms, are here included in maintenance. *B* is known to scale with body mass (*M*) as a power law and temperature (*T*), measured in grams and kelvins respectively, according to the equation:

(1) $$B=B_{0}M^{3/4}e^{-E/kT}$$

where *B*_0_ is a taxon-specific normalisation constant, *M*^3/4^ is the scaling with body mass, *e*^−*E/kT*^ is the exponential Arrhenius function, *E* is the activation energy, *k* is the Boltzmann's constant (8.62 × 10^−5^ eV K^−1^) (Table 1) (Peters, 1983; Gillooly et al., 2001; Brown and Sibly, 2012). In what follows it is sometimes convenient to consider effects of temperature relative to a reference temperature, *T*_*ref*_. The effect of temperature is then given by $e^{\left(-E/k)((1/T)-(1/T_{ref})\right)}$.

##### 2.1.7.2 Ingestion and energy uptake

Variation in food density affects the rate of ingestion of food up to an asymptote according to a type II functional response (Holling, 1959; Ricklefs and Miller, 2000), so that:

$$\text{ingestion rate}\propto \frac{X}{h+X}$$

where *X* is food density (g/0.01 m^2^) and *h* is a constant that shows how quickly the response curve reaches its maximum as food density increases. Ingestion rate is also proportional to the surface area (*M*^2/3^) of an individual as the search rate depends on the food gathering apparatus (Kooijman and Metz, 1984; Pilarska, 1977) and to temperature, giving:

(2) $$\text{ingestion rate}=IG_{\max}e^{\left(-E/k)((1/T)-(1/T_{ref})\right)}\frac{X}{K+X}M^{2/3}$$

where *IG*_max_ is the maximum ingestion rate recorded of a 1-g *E. fetida* under optimal feeding conditions (g/day/g) (Table 1). Ingestion rate is measured in g/day and this is converted into kJ/day depending on the energy content of the food. After ingestion, food is processed by the digestive system and a proportion, assimilation efficiency, becomes available for allocation to the various functions shown in Fig. 1. The value of the assimilation efficiency (*A*_e_) (Table 1) depends on diet but not body mass (Hendriks, 1999).

##### 2.1.7.3 Growth

After expenditure to maintenance and, at the adult stage, to reproduction, individuals allocate remaining energy to somatic growth. The maximum growth rate of an individual under optimal conditions is assumed to follow the von Bertalanffy (1957) growth equation:

(3a) $$M=M_{m}{\left(1-\left(1-{\left(\frac{M_{b}}{M_{m}}\right)}^{1/3}\right)e^{-r_{B}t/3}\right)}^{3}$$

where *M*_*b*_ and *M*_*m*_ denote mass at birth and maximum mass respectively and $r_{B}$ is the Bertalanffy growth constant, obtained by fitting Eq. (3a) to data recording the increase in individual biomass over time under optimal conditions. The maximum growth rate per time-step is obtained from (Sibly et al., 2013):

(3b) $$\Delta M=r_{B}e^{\left(-E/k)((1/T)-(1/T_{ref})\right)}(M_{m}^{1/3}M^{2/3}-M)$$

The energy costs of growth are determined from the new mass calculated from Eq. (3b) and the energy costs of production (*E*_*c*_ + *E*_*s*_) (Table 1). Eq. (3b) shows how the maximum rate at which resources can be allocated to growth changes as an individual increases in mass. If insufficient energy is available to support maximal growth, growth rate is reduced accordingly.

##### 2.1.7.4 Reproduction

Reproduction is assumed to take priority over growth in adults, because in the absence of a sexual partner, earthworms grow larger (Neuhauser et al., 1980). Energy allocated to reproduction by adults goes directly to the production of an egg until oviposition inside a cocoon (note this is a slight simplification since *E. fetida* can insert more than one egg into a cocoon). The maximum rate of energy allocation to reproduction per day increases linearly with adult mass (Mulder et al., 2007):

(4) $$\Delta R=r_{m}e^{\left(-E/k)((1/T)-(1/T_{ref})\right)}M$$

where *r*_*m*_ is the maximum rate of energy allocation to reproduction per unit of adult mass (kJ/g/day). The energy cost of producing a hatchling is *M*_*c*_ (*E*_*c*_ + *E*_*s*_) (Table 1) and the hatchling's energy reserve content is initially *M*_*c*_ *E*_*c*_, which is utilised for maintenance during the incubation period.

##### 2.1.7.5 Energy reserves and starvation

If any assimilated energy remains after expenditure on relevant life processes (Fig. 1) it is stored in an individual's energy reserves. Energy is stored as glycogen (Byzova, 1977), costing *E*_s_ = 3.6 kJ to store 1 g with an energy content of *E*_c_ = 7 kJ (Sibly and Calow, 1986; Peters, 1983). When energy is not available from ingested material, maintenance costs are taken from energy reserves, allowing individuals to survive for some time under starvation (Sousa et al., 2010; Gunadi et al., 2002). Furthermore, as evidence supports the assumption that reproduction continues even when food is limiting (Reinecke and Viljoen, 1990), the energy reserves are assumed to be utilised for reproduction above a threshold of 50% of an individual's maximum energy reserves, taken as $(M/2)E_{c}$ (e.g. Peters, 1983). If food limitation continues and the energy reserves decline below 50% of an individual's maximum energy reserves, individuals are considered to be in a state of starvation. Under these conditions tissue is catabolised to cover maintenance costs, resulting in net weight loss (Gunadi and Edwards, 2003); individuals die if their mass falls to that at birth (*M*_*b*_) following Reinecke and Viljoen (1990).

##### 2.1.7.6 Movement

On the basis that Kobetičová et al. (2010) found movement in *E. fetida* individuals to be random, we modelled individual movements as random in direction from a uniform distribution between −90° and 90° and distance travelled as 5 cm per time-step.

##### 2.1.7.7 Survival

The survival of individuals living in field populations is determined by the availability of energy resources to maintain life cycle processes together with temperature and soil moisture specific mortality rates. Individuals die of starvation if their energy resources are depleted, and additional mortality rates were imposed using a regression equation derived from Presley et al. (1996):

(5) $$\text{mortality rate}(\%)=12.7-0.0010\;SM-0.0861\;T+0.000009\;SM^{2}+0.000147\;T^{2}$$

where *SM* is soil moisture (%) and *T* is soil temperature (K). Individual adults and juveniles die according to Bernoulli processes with daily mortality rates given in Eq. (5).

### 2.2 Model simulations

#### 2.2.1 Laboratory experiments

The model was set up to simulate the conditions of published laboratory experiments to evaluate model fits to growth and reproduction data. The studies of Gunadi et al. (2002), Gunadi and Edwards (2003) and Reinecke and Viljoen (1990) were used to evaluate growth and reproduction. Details of model initialisation for these simulations are summarised in Table 2. Gunadi and Edwards (2003) recorded a mortality rate of 28% before 161 days and added 10 of the surviving adults to a new substrate on day 161, which is simulated in the model as shown in Table 2. In the case of Reinecke and Viljoen (1990) individuals were aged 25 days at the beginning of the experiment (Reinecke, AJ, Stellenbosch University, South Africa, pers. comm.) and the model simulations were run accordingly. To assess the model's ability to capture chemical effects on the sublethal endpoints growth and reproduction, we used data from Helling et al. (2000) and Maboeta et al. (2004) to investigate the effects of copper oxychloride, and those of Zhou et al. (2007, 2011) for chlorpyrifos. Food quantities were uniformly distributed over the landscape at the feeding times indicated in Table 2.

#### 2.2.2 Field studies

The model was used to simulate *E. fetida* population dynamics under the field conditions studied by Monroy et al. (2006). The authors collected data from a manure heap exposed to seasonal environmental conditions over one year. Autumn, winter, spring and summer rainfall (mm)/ambient temperature (°C) were recorded as 600/9.1, 200/8.1, 770/12.1 and 250/16, respectively, but the detailed environmental data needed for our model were not reported and therefore had to be estimated as follows. We followed Meyer's (1926) suggestion that soil moisture content is equivalent to precipitation (mm) divided by the saturation deficit (mm Hg) of air, and assumed an average annual humidity of 50%, giving a saturation deficit of 20 mmHg (Frankham et al., 2004). Manure was assumed to have a 30% higher water holding capacity than soil, following Unger and Stewart (1974). Petersen et al. (1998) found large cattle manure heaps to be 2.3–6.2 °C warmer than the surrounding air at a depth of 50 cm, with a mean air temperature of 10 °C, and on this basis we assumed a compositional warming effect of 5 °C for ambient temperatures reported over 10 °C as the manure heap in Monroy et al. (2006) is described as “a temporary heap of cow manure from a small farm”. These calculations give soil moisture (%)/soil temperature (°C) values of 60/9, 40/8, 70/17 and 45/21 for autumn, winter, spring and summer respectively. To mimic natural variation these values were varied by drawing each day from a normal distribution with standard deviation (SD) 5 °C for temperature and 10% for moisture. As food density was not measured in the original study estimates of seasonal availability were made as follows. Maximum quantities of 350 kg manure/heap were reported by Rufino et al. (2007) for large dairy farms. As the manure heap in Monroy et al. (2006) was only a temporary heap on a small farm measured per m^2^ rather than per heap, the maximum quantity of manure was taken as 50 kg/m^2^. Seasonal variation was estimated on the basis that more cattle feed is provided during winter and spring, that decomposition rates increase in summer, and that most of the manure heap was removed in late spring each year (Monroy et al., 2006), yielding values of 10, 15, 50 and 5 kg/m^2^ of manure for autumn, winter, spring and summer respectively. To account for spatial heterogeneity landscape patches were replenished with food supplies taken at random from normal distributions with these mean values and SD 10% of the mean. Although these are mere approximations they are the best estimates available.

### 2.3 Incorporating toxicity data

**Fig. 3:**
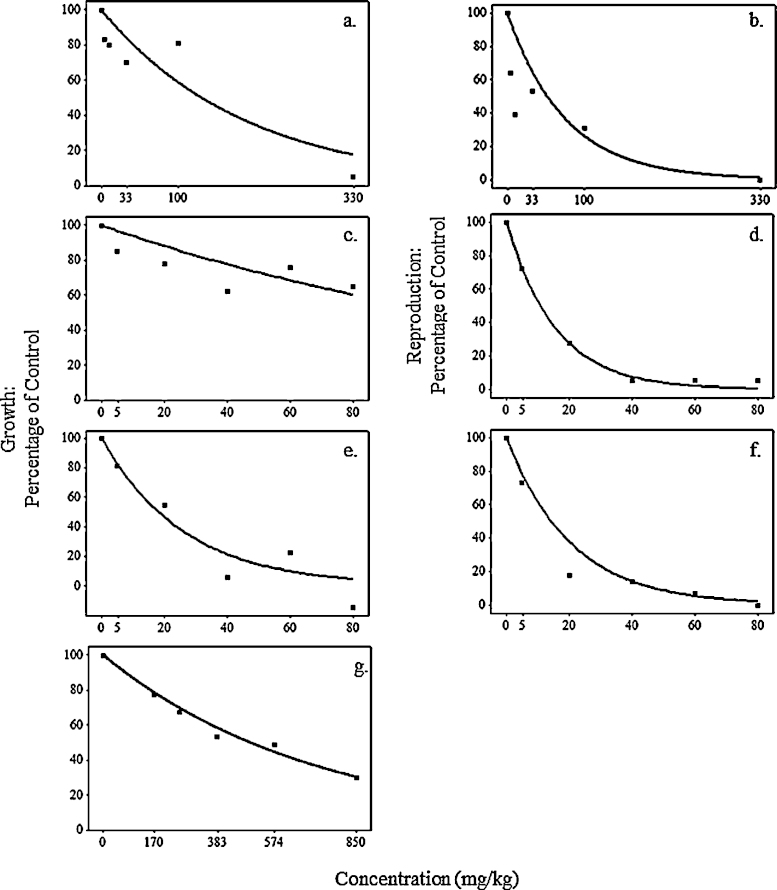
Modelling dose–response curves. Curves fitted to experimental laboratory data for (a, c, e and g) growth and (b, d and f) reproduction, for (a, b) copper oxychloride by Helling et al. (2000); (c, d) chlorpyrifos by Zhou et al. (2007), (e, f) chlorpyrifos by Zhou et al. (2011) and (g) copper oxychloride by Maboeta et al. (2004). *R*^2^ values for regression curves in a, b, c, d, e, f and g are: 0.81, 0.73, 0.65, 0.99, 0.92, 0.96 and 0.99, respectively. Reproduction and growth data are represented as a reduction in life cycle trait compared to the control under different concentrations. Regression coefficients determining these curves are used to investigate the putative metabolic pathway for each pesticide.

We used experimental literature data on the sublethal effects of chlorpyrifos and copper oxychloride on *E. fetida* recorded in the laboratory (Table 2) to model dose–response curves at the metabolic level. Pesticide risk assessments typically convert dose–response relationships between chemical concentration and mortality, into linear relationships using logit or probit transformations. As the data available here do not result in linear relationships, an alternative procedure was necessary. Individual biomass and cocoon production values for different treatment concentrations in each case study were converted to percentages of the control value. The data were then generally well fitted by exponentially declining curves, of the form:

(6) $$R(C)=e^{kC}$$

where *R*(*C*) is % trait compared to control, *k* is a chemical-specific coefficient calculated by regressing log (% trait compared to control/100) against chemical concentration (*C*) in mg/kg. Eq. (6) represents the dose–response relationship between chemical concentration and a life cycle trait (growth or reproduction), presented in Fig. 3, but does not specify which physiological parameter was affected.

To identify the physiological processes affected we investigated the various possibilities in which either one or two of the processes shown in Fig. 1 were affected, here called toxicity submodels. Inspection of Fig. 1 indicates that chemicals can affect ingestion, assimilation, maintenance, growth or reproduction, the rates of which are governed by physiological parameters $IG_{\max},A_{e},B_{0},r_{B}$ or *r*_*m*_, respectively (Table 1). Varying $IG_{\max}$ has the same effect as varying $A_{e}$; here we only consider the former. Varying *IG*_max_ or increasing *B*_0_ alone would have effects on both growth and reproduction as the energy available for expenditure to these processes is reduced. We do not consider varying either *r*_*B*_ or *r*_*m*_ alone as immediate effects would be on either growth or reproduction, not both together as indicated by the data in Fig. 3. The plausible submodels in which just one or two processes are affected are shown in Table 3. Each submodel supposes that the chemical-specific toxicity coefficient (*k*) obtained by fitting Eq. (6) to the data shown in Fig. 3 determines the relationship of the chemical concentrations to physiological parameters $IG_{\max},r_{m},r_{B}\;or\;B_{0}$, so that *R*(*C*) in Eq. (6) is % parameter value compared to control. Effects of chemical exposure on life cycle traits (growth, reproduction) are then identified by model simulations. For example, in simulating Helling et al.’s (2000) experiment with submodel T2 the parameter values of *IG*_max_ and *r*_*B*_ follow the dose-response curves in Fig. 3a and b, respectively. In submodel T4 we supposed the toxin led to an increase in the maintenance parameter $B_{0}$, either to eliminate/detoxify the toxin, or to repair damage. Here we assumed that above a concentration of 100 mg/kg there is a linear relationship between $B_{0}$ and *C* so that:

$$\begin{array}{l} B_{0}=B_{0\;\text{control}},\;\text{if}\;C\leq 100;\\ B_{0}=B_{0\;\text{control}}\times 0.01\;C,\;\text{if}\;C>100. \end{array}$$

The toxicity submodel producing the best fit to growth and reproduction data in each case study was calculated to identify the most plausible underlying physiological effects of toxicity. To evaluate model outputs using experimental data we used a likelihood approach by calculating Akaike information criterion (AIC) values for measuring the relative goodness of fit of each model (Anderson, 2008):

(7) $$\text{AIC}c=n\;\log({\hat{\sigma }}^{2})+2n^{\prime }\left(\frac{n}{n-n^{\prime }-1}\right)$$

where ${\hat{\sigma }}^{2}=\Sigma {\hat{\varepsilon }}_{i}^{2}/n$ and ${\hat{\varepsilon }}_{i}$ are the normalised differences between the experimental data and the model outputs, *n* is the sample size and *n*′ is the number of parameters, here represented by the number of toxicity coefficients used in the simulations. The differences between submodel ($\Delta_{i}$) were then calculated as: $\Delta_{i}=\text{AIC}c_{i}-\text{AIC}c_{\min}$ where $\text{AIC}c_{\min}$ is the best performing model. Full details of the calculations are available in Appendix A.

### 2.4 Model evaluation

The model was thoroughly tested to verify that it behaved as expected. Here we present only local sensitivity analysis; further evaluation methods used are described in the TRACE document in the supplementary material.

#### 2.4.1 Local sensitivity analysis

The sensitivity of the model to the values of its parameters is presented in Table 4. The model was run with the parameter values of Table 1 (*N* = 100) and again with parameter values increased one at a time by 10% (*N* = 100). Changes in model outputs (adult biomass, juvenile biomass and cocoons produced per adult) are shown in Table 4. Also shown in Table 4 is the sensitivity of the model to the baseline values of the environmental variables varied individually; these were soil temperature: 25 °C; soil moisture: 60%; and food density: 20 g per patch. All simulations were run for one year under the field study conditions outlined in Section 2.2.2.

## 3 Results

All data used in model evaluation are included together with model outputs in supplementary material (Appendix B).

### 3.1 Local sensitivity analysis

Table 4 shows the sensitivity of adult biomass, juvenile biomass and reproductive output to 10% changes in model parameters. All output variables were most sensitive to parameters affecting temperature relationships, with activation energy (sensitivities 1.09–1.18), the reference temperature (−0.95 to 1.42) and soil temperature (−0.52 to 0.48) having most impact. These results show the importance of temperature for earthworm population dynamics.

### 3.2 Individual life cycle processes

**Fig. 4:**
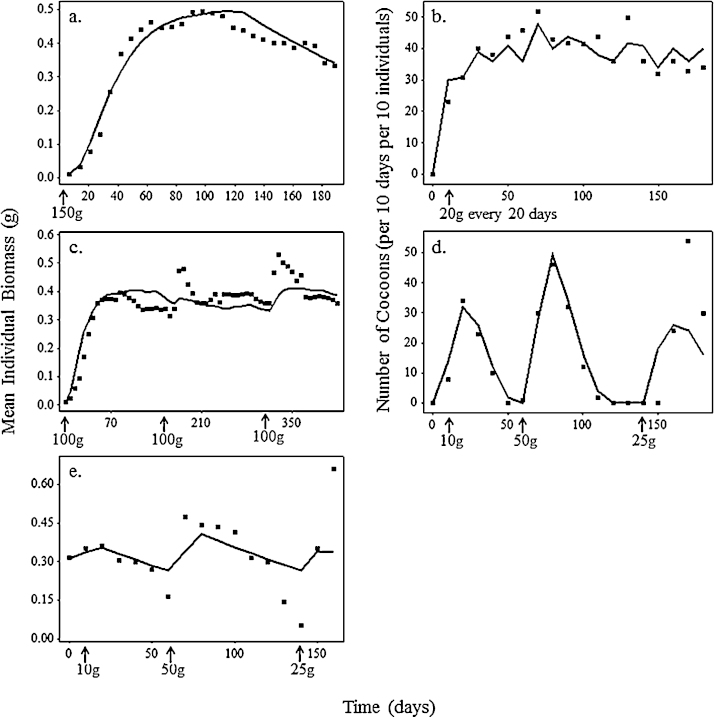
Comparison between model outputs (lines) and recorded growth (a, c and e) and reproduction (b and d) data (points) from (a) Gunadi et al. (2002), (b) Gunadi and Edwards (2003) and (c–e) Reinecke and Viljoen (1990). Arrows indicate the time and amounts of food supplied.

In this section we present life cycle data for *E. fetida* from experimental studies together with the outputs of model simulations run under the same conditions. The results for growth and reproduction under control condition are shown in Fig. 4. Note that the model predictions shown in Fig. 4 were obtained without fitting parameters to data. All parameter values were obtained from the literature as shown in Table 1. Survival in all experiments and model simulations, except for the case of Gunadi and Edwards (2003), was 100%.

Simulation of the Gunadi et al. (2002) experiment showed a good match to data in both the increasing phase (optimal food) and the descending phase when individuals lost body mass because the food supply was depleted (Fig. 4a). Model predictions of mass loss during starvation are less accurate in Figs. 4c and e, but the discrepancies are in opposite directions, so it would not be possible to fit both datasets well. Reinecke and Viljoen (1990) recorded the reproduction rate of *E. fetida* under optimal and limiting conditions (Figs. 4b and d), with model outputs fitting the experimental data under both conditions well.

### 3.3 Sublethal effects on growth and reproduction

The toxicity submodels shown in Table 3 were simulated for each literature experiment by altering the physiological parameters according to the dose–response relationships of Fig. 3 under the conditions outlined in Table 2. Model fits to the literature data at the tested concentrations were then evaluated using a likelihood method described in full in the supplementary material. The fits of the toxicity submodels to the data were assessed by Δ_*i*_ and these values together with evidence ratios (ERs) (see e.g. Anderson, 2008) are given in Table 5. When adequate food was provided (i.e. where food was not a limiting factor) the toxic effects of both copper oxychloride and chlorpyrifos on growth and reproduction were best described by supposing physiological parameters *r*_*m*_ and *r*_*B*_ were directly affected, using toxicity submodel T3 (Table 5). When food was limited, in the experiment of Maboeta et al. (2004), toxicity models T1–T3 resulted in individuals remaining at their starting biomass throughout, which is why the Δ_*i*_ values for models T1–T3 in Table 5 are near equal. Only when supposing the parameter *B*_0_ was affected in toxicity submodel T4 did the model provide a good fit to the data. Table 5 shows that the odds against toxicity models other than the best performing (T3 or T4) being better are very high, given by the evidence ratios, in each case >10^6^:1.

**Fig. 5:**
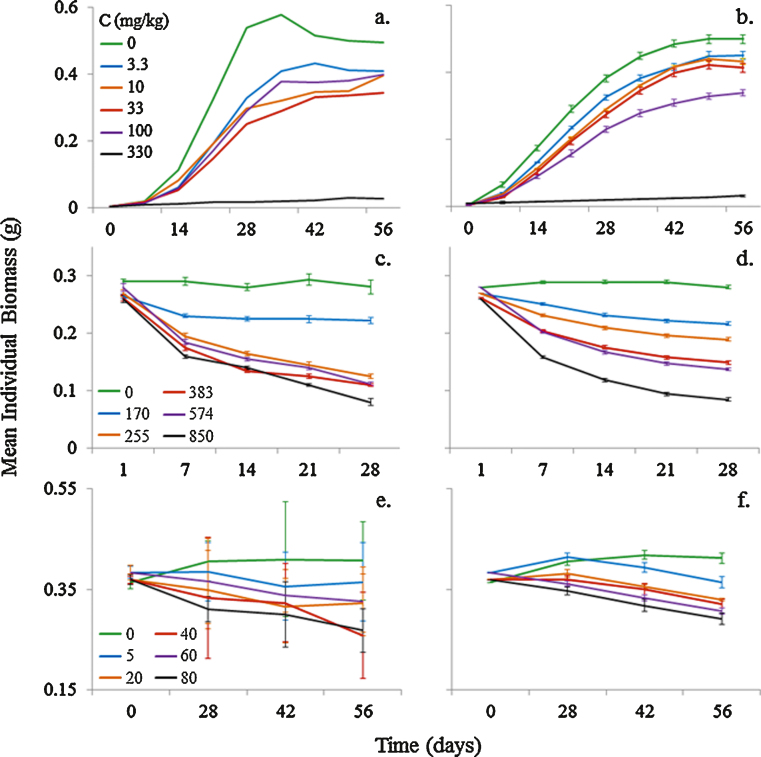
Comparison of experimental toxicity data (left-hand panels) and model simulations of toxicity experiments (right hand panels). (a, b) the effects of copper oxychloride (Helling et al., 2000) modelled using submodel T3; (c, d) copper oxychloride (Maboeta et al., 2004) using T4; and (e, f) chlorpyrifos (Zhou et al., 2007) using T3.

**Fig. 6:**
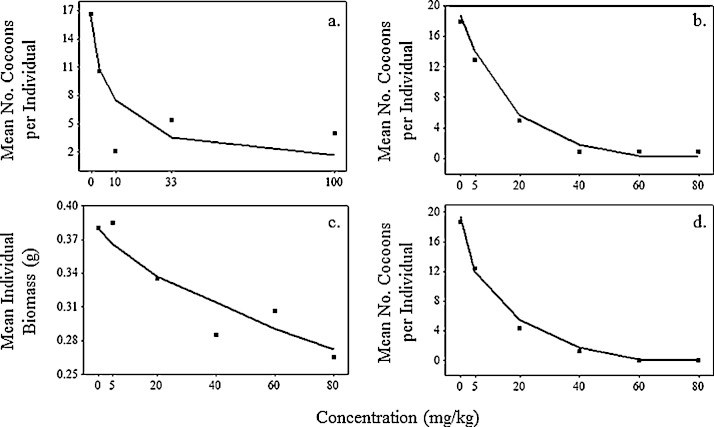
Comparison of model simulations (lines) with data on reproduction from (a) Helling et al. (2000); (b) Zhou et al. (2007) and (d) Zhou et al. (2011). Panel (c) shows the fit for growth data in Zhou et al. (2011). Chemicals were chlorpyrifos (a, c and d) and copper oxychloride (b).

Figs. 5 and 6 show growth and reproduction data for *E. fetida* from experimental studies under various exposures of copper oxychloride and chlorpyrifos together with the outputs from the best performing toxicity submodel simulations run under the same conditions. Simulation of the Helling et al. (2000) experiment shows good model fits to growth data (Fig. 5a and b) and reproduction data (Fig. 6a) under control and maximum concentrations, although at intermediate concentrations experimental responses do not increase monotonically with concentration. However these results are generally well predicted by submodel T3 in which the parameters controlling allocation of energy to growth and reproduction are directly affected. Effects of copper oxychloride on growth in Maboeta et al. (2004) (Fig. 5c) were not explained by imposing stress on physiological parameters directing the allocation of energy ($IG_{\max},r_{B},r_{m}$). As the authors in this case study gave a high density of 20 adult *E. fetida* a limited supply of food at the beginning of their experiment there were minimal changes in biomass in the control treatment, indicating that energy ingestion was restricted. The data shows an increase in weight loss with chemical concentration, explained by our energy budget model as the catabolisation of tissue for increasing maintenance requirements. This mechanism is described by submodel T4, resulting in the model outputs presented in Fig. 5d which capture the span of the response. Growth data presented by Zhou et al. (2007) (Fig. 5e) shows great variation in individual biomass between treatment concentrations of chlorpyrifos, with the standard errors for each treatment overlapping. Yet, based on the mean biomasses recorded the model provides a reasonable fit to the growth data (Fig. 5f) and a good fit to the reproduction data (Fig. 6b).

Zhou et al. (2011) provided the same experimental conditions as Zhou et al. (2007) and recorded mean individual biomass and cocoon production after 56 days exposure as shown in Fig. 6c and d. Submodel T3 again provides a good fit to the data.

### 3.4 Population dynamics in the field

**Fig. 7:**
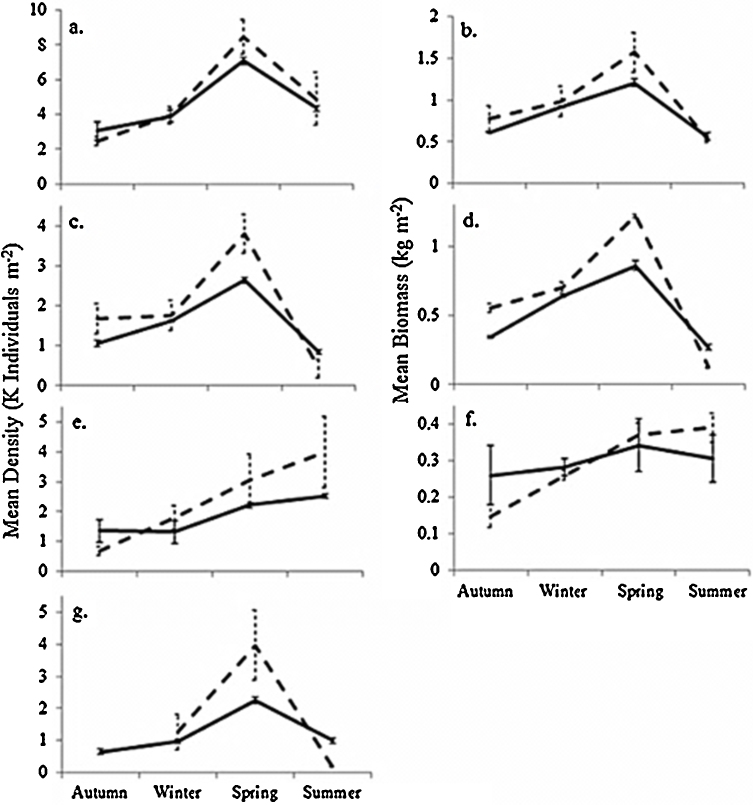
Comparison between (left-hand panels) field population density data and (right-hand panels) population biomass data from Monroy et al. (2006) (dashed line) and model simulations (solid lines) with standard errors. (a, b) Total population; (c, d) adults; (e, f) juveniles; (g) cocoons. Juveniles here comprise the hatchlings, juveniles and preclittelates that were counted separately in the field.

Population data reported by Monroy et al. (2006) under field conditions are compared with mean results from ten-year-long simulations of the Monroy et al. (2006) study in Fig. 7.

Patterns of seasonal changes in population density and biomass (Figs. 7a and b) are generally well predicted by the model, although adult density and biomass and cocoon density are slightly underestimated in spring. The higher cocoon densities observed in spring may be due to higher temperatures occurring within the manure heap under high population densities, not considered in the model.

## 4 Discussion

Key aspects of earthworm population ecology have been realistically simulated using the simple energy budget-driven ABM developed and evaluated in this paper. The distinctive features of our approach are that we have aimed to produce a minimal model in which (1) each individual has its own energy budget operating according to accepted principles of physiological ecology; (2) components of the model are parameterised using literature data at the level at which it is generally available, the level of the individual; (3) individuals live in a spatially explicit landscape so that food availability depletes in those areas in which individuals forage; (4) individuals interact with others in natural ways, they meet others to mate but compete for food with others in their patch; (5) the combination of energy budget and ABM approaches mechanistically relates individual physiology to ecologically relevant conditions. Good model fits were obtained to published experimental data for individual growth and reproduction (Fig. 4) under both optimal and limiting feeding conditions, and to population data in the field (Fig. 7). Model applications to toxicology experiments provide a useful basis for interpreting how pesticides achieve their effects, with good model fits under exposures of chlorpyrifos and copper oxychloride in Figs. 5 and 6. It is important to note that these fits were obtained without modifying parameter values. Parameter values were obtained from the literature and are shown in Table 1.

Essential to achieving the good model fits presented is the accurate representation of physiological mechanisms and the inclusion of depleting food supplies. The timing of starvation in Figs. 4a, c and e was well predicted by the model when food supplies varied. As food depletes from the ABM landscape at a rate proportional to individual ingestion rates, the timing of starvation supports our assumptions about energy acquisition, expenditure and the onset of starvation. The over and under-prediction of maximum body mass in Figs. 4c and e, respectively, may be related to the energy content of the food, as the quality of cow manure differs with decomposition stage. These discrepancies highlight the need for comprehensive environmental measurements in experimental work. The effect of decreasing food availability on life cycle processes is particularly evident in the data of Reinecke and Viljoen (1990) (Figs. 4b, d and e). Under varying food availability, reproduction continued during periods of individual weight loss (i.e. starvation). Aiding the good model fits to the timing of weight loss and reproduction rates under limiting conditions are the assumptions made about the priorities of energy allocation: adults continue to allocate energy to reproduction at the expense of growth, until a critical low energy reserve threshold is reached. The response of our model is supported by Klok's (2007) study of *L. rubellus* in limiting conditions: earthworms reduced their individual growth rates but maintained reproduction as population density was increased. Under limiting and fluctuating conditions, such as those likely to occur in the field, it is important that life cycles are modelled realistically.

Information about food supplies was essential in the identification of the physiological parameters affected by pesticides. When earthworms are provided with sufficient food, the case studies of Helling et al. (2000) for copper oxychloride and Zhou et al.’s (2007, 2011) for chlorpyrifos indicate that reproduction is more sensitive than growth to toxic stress (Table 3; Figs. 5 and 6). To understand the underlying physiological mechanisms of toxicity it is necessary to investigate alternative models of how toxic chemicals may achieve their effects. When sufficient food was available, the model best fitting the data was T3 (Table 5), which supposes that stress is imposed directly on the physiological processes of reproduction and growth. Svendsen and Weeks (1997) found copper exposure to increase food consumption by *Eisenia andrei* up to concentrations of 80 mg/kg, which had proportionally positive effects on individual growth. In contrast reproduction was reduced from exposures of 20 mg/kg and completely inhibited at 160 mg/kg whilst growth persisted at a reduced rate. Many other experimental studies have also identified reproduction to be more sensitive to toxicants than growth when sufficient food is available for *E. fetida* (Robidoux et al., 2004; Gunn and Sadd, 1994) and field populations of *Lumbricus rubellus* (Svendsen et al., 2007). These findings are in line with our interpretation of the physiological response of *E. fetida* to the two pesticides tested in this paper.

By contrast when little food was available as in the experiment of Maboeta et al. (2004), maintenance was fuelled from energy reserves and the animals lost weight (Fig. 5c and d). Similar effects have been observed in biomarker studies of earthworms, in which an inhibition of AChE activity in *E. fetida* during acute toxicity tests resulted in self-digestion (Rao et al., 2003). Physiologically this suggests that AChE inhibition led to muscular paralysis at higher concentrations (e.g. Gupta and Sundararaman, 1991), impeding food consumption, while at the same time energy was required to eliminate the toxicant (Calow, 1991). As in Maboeta et al. (2004) our energy budget model describes the underlying physiological processes of these observations as catabolisation of tissue under starvation, with rates of weight loss proportional to the increase in maintenance rates. This emphasises the usefulness of building and comparing models of how toxic chemicals achieve their effects in helping to interpret toxicology test results. As feeding conditions are variable in field conditions we suggest the simple combination of the two best performing toxicity submodels (T3 and T4) for population level simulations.

Density dependence in our ABM is an emergent property of the modelled interactions between individuals and their food supply (Grimm and Uchmanski, 2002; Sibly et al., 2009). As fluctuations in food availability are the rule in the field, understanding of how food density affects population dynamics through its effects on individual growth and reproduction is of great importance. Uvarov and Scheu (2004) suggested that density-dependent regulation of earthworm populations results directly from a decrease in food availability at higher densities. Many authors have also observed the negative effect of high population densities on *E. fetida* growth (Hartenstein, 1984), sexual maturation (Neuhauser et al., 1980) and reproduction (Kammenga et al., 2003). At the population level, our model accurately predicted how seasonal changes of just three environmental state variables (food density, soil temperature and soil moisture) determine earthworm population structure (Fig. 7). In spring cocoon densities were higher than predicted, perhaps due to higher temperatures when population densities were high. Reproduction is particularly affected by temperature and many authors have noted a highly seasonal occurrence of cocoon production (Gerard, 1967; Evans and Guild, 1948; Whalen et al., 1998). The high cocoon density observed in spring by Monroy et al. (2006) was linked to a decrease in individual adult biomass, a pattern that the model reproduces due to preferential allocation of energy to reproduction before growth (Fig. 7d and g). Overall the model captures the major aspects of the population dynamics recorded by Monroy et al. (2006) but would have benefited from more detailed recordings of abiotic factors such as soil temperature and organic matter content being reported. The ability of the model to replicate field observations suggests it incorporates a realistic representation of underlying metabolic processes.

The development of ABMs incorporating individual energy budgets is essential for realistic modelling of populations regulated by environmental conditions. The capacity of the model to capture the linkage between food density and metabolism results from the ability of ABMs to model individual–landscape interactions, and illustrates the applicability of these methods to realistic chemical risk assessments at the laboratory scale. For these purposes our model provides a promising alternative to simpler more deterministic methods. An advantage of ABM modelling is that it readily extrapolates to larger scales, and prediction at the scale of agricultural fields should be immediately possible. Field application rates of agricultural chemicals tend to be much lower than those tested in lower tier risk assessments, but ABMs like ours based on physiological knowledge at the individual level can give insights into how sublethal concentrations affect populations. As an example, consider the findings of Senapati et al. (1992); application of malathion led to a dominance of adults in a field population which the authors interpreted as being caused by an increase in individual costs of maintenance. In our energy-budget-driven ABM, these increased costs of maintenance would slow growth in juveniles and reduce reproduction rates of adults, and so support Senapati et al.’s interpretation. We do not provide extrapolations to pesticide application effects on *E. fetida* field populations here because no experimental data exists for validation, as *E. fetida* is not a wide-spread natural field species (Paoletti, 1999). Nevertheless good model fits to field population data under control conditions in Fig. 7 are an encouraging basis for further work extending the model to more ecologically relevant species. Taking account of the heterogeneous distribution of chemicals in the soil environment and of their degradation with time will then allow the evaluation of model predictions at the field scale. Such a model could help extrapolate from laboratory to field conditions and from one set of field conditions to another or from species to species.

## Figures and Tables

**Table 1:** Default parameter values for the energy budget model with reference to literature data sources.

Symbol	Definition	Value	Unit	Reference	Notes
*A*_*e*_	Assimilation efficiency	0.50	–	Hobbelen and van Gestel (2007)	p. 376 (see Appendix A)
*B*_*o*_	Taxon-specific normalisation constant	967	kJ/day	Meehan (2006)	Calculated from Table 2, p. 881 and Eq. (4) (see Appendix A)
*E*	Activation energy	0.25	eV	Meehan (2006)	p. 880
*E*_*c*_	Energy content of tissue	7	kJ/g	Peters (1983)	p. 235
*E*_*s*_	Energy cost of synthesis	3.6	kJ/g	Sibly and Calow (1986)	Calculated from p. 54 to 55
*E*_*x*_	Energy content of food	21.2	kJ/g	Wang et al. (2011)	p. 173
*IG*_max_	Maximum ingestion rate	0.70	g/day/g	Neuhauser et al. (1980)	Derived/re-calculated from Figure 6, p. 96 (see Appendix A)
*h*	Half saturation coefficient	3.5	g/0.01 m^2^	Neuhauser et al. (1980)	
*M*_*b*_	Mass at birth	0.011	g	Gunadi et al. (2002)	Derived from Table 1, p. 18 and Figure 1, p. 19
*M*_*c*_	Mass of cocoon	0.015	g	Hartenstein et al. (1979)	Derived mean from Figure 5, p. 333
*M*_*p*_	Mass at sexual maturity	0.25	g	Gunadi et al. (2002)	Derived from Table 1, p. 18 and Figure 1, p. 19
*M*_*m*_	Maximum asymptotic mass	0.50	g	Gunadi et al. (2002)	
*r*_*B*_	Growth constant	0.177	day^−1^	Gunadi et al. (2002)	Figure 1, p. 19 fitted to Eq. (5a) (see Appendix A)
*r*_*m*_	Maximum rate of energy allocation to reproduction	0.182	kJ/g day	Tripathi and Bhardwaj (2004)	Derived from p. 281 (see Appendix A)
*T*_*0*_	Incubation period	23	Days	Reinecke et al. (1992)	Table 3, p. 1298
*T*_*ref*_	Reference temperature	298.15	kelvins	Tripathi and Bhardwaj (2004)	p. 280

**Table 2:** Experimental conditions used in model simulations for comparison with growth and reproduction data from the named studies. *N* gives the number of replicated simulations. Soil moisture content was 80% in all experiments.

Study	*N*	No. individuals	Food quantity (g)	Feeding times (days)	Landscape size (m^2^)	*T* (°C)
Gunadi et al. (2002)	3	5	150	0	0.08	20
Gunadi and Edwards (2003)	4	8, 6/5, 10	100	0, 161 and 315	0.08	20
Reinecke and Viljoen (1990)	4	10	10, 50 and 25	10, 60 and 140	0.04	25
Reinecke and Viljoen (1990)	4	10	20	Per 20 days	0.04	25
Reinecke and Viljoen (1990)	4	10	10, 50 and 25	10, 60 and 140	0.04	25
Helling et al. (2000)	4	10	75, 30, 30, and 30	0, 35, 42, 49	0.06	25
Maboeta et al. (2004)	3	20	0.54	0	0.08	25
Zhou et al. (2007)	4	10	5	0, 28	0.08	20
Zhou et al. (2011)	4	10	5	0, 28	0.08	20

**Table 3:** Tested toxicity submodels used to identify the physiological pathways disrupted by pesticides. In each case the specified physiological parameters were affected according to dose-response curves parameterised as in Fig. 3. $IG_{\max}$ is maximum ingestion rate, $r_{m}$ is maximum rate of energy allocation to reproduction, $r_{B}$ is the von Bertalanffy growth constant and $B_{0}$ is a taxon-specific normalisation constant used for calculating maintenance rates.

Toxicity submodel	Parameter	Predicted observations in adult life cycle traits
T1	$IG_{\max}$	Growth more reduced than reproduction
T2	$IG_{\max}\;\text{and}\;r_{m}$	Growth and reproduction similarly reduced
T3	$r_{m}\;\text{and}\;r_{B}$	Reproduction more reduced than growths
T4	$B_{0}$	Growth more reduced than reproduction or accelerated weight loss under resource limitation

**Table 4:** Sensitivity analysis showing ratio of % changes in mean output variables to 10% changes in parameter values, with standard errors. Thus, sensitivities between −1 and +1 represent changes in outputs between −10% and +10% of baseline values, respectively.

Parameter	Output variables		
	Adult biomass	Juvenile biomass	Cocoons per adult
Assimilation efficiency (*A*_*e*_)	0.03 ± 0.08	0.02 ± 0.14	0.04 ± 0.09
Taxon-specific normalisation constant (*B*_*o*_)	0.12 ± 0.10	0.09 ± 0.08	0.18 ± 0.14
Activation energy (*E*)	1.09 ± 0.31	1.18 ± 0.19	1.17 ± 1.11
Energy content of tissue (*E*_*c*_)	0.12 ± 0.11	−0.41 ± 0.19	0.04 ± 0.12
Energy cost of synthesis (*E*_*s*_)	0.13 ± 0.11	−0.01 ± 0.03	−0.06 ± 0.12
Energy content of food (*E*_*X*_)	−0.06 ± 0.04	0.08 ± 0.07	0.11 ± 0.08
Maximum ingestion rate (*IG*_max_)	−0.26 ± 0.09	0.01 ± 0.09	−0.10 ± 0.11
Half saturation coefficient (*h*)	0.01 ± 0.02	0.01 ± 0.06	0.03 ± 0.05
Mass at birth (*M*_*b*_)	−0.02 ± 0.06	−0.01 ± 0.16	−0.10 ± 0.04
Mass at sexual maturity (*M*_*p*_)	0.07 ± 0.09	0.26 ± 0.11	0.01 ± 0.09
Maximum asymptotic weight (*M*_*m*_)	0.03 ± 0.07	0.35 ± 0.12	−0.05 ± 0.10
Mass of cocoon (*M*_*c*_)	0.19 ± 0.13	−0.08 ± 0.01	−0.10 ± 0.18
Growth constant (*r*_*B*_)	−0.02 ± 0.10	0.08 ± 0.12	0.08 ± 0.10
Maximum rate of energy allocation to reproduction (*r*_*m*_)	0.02 ± 0.07	0.01 ± 0.09	0.03 ± 0.01
Incubation period (*T*_0_)	0.02 ± 0.03	0.01 ± 0.10	−0.02 ± 0.08
Reference temperature (*T*_*ref*_)	1.42 ± 0.13	−0.95 ± 0.13	1.03 ± 1.04

Environmental variable			
Soil temperature (*T*)	0.48 ± 0.15	−0.52 ± 0.09	0.25 ± 0.22
Soil moisture (*SM*)	0.01 ± 0.10	0.01 ± 0.08	0.02 ± 0.01
Food density (*X*)	−0.17 ± 0.08	0.13 ± 0.09	−0.09 ± 0.03

**Table 5:** Comparison of toxicity submodel (T1–T4) fits to the experimental data. AIC_c_ differences (Δ_*i*_) between toxicity submodels and evidence ratios (*ER*) indicate the relative fit to the data. Higher Δ_*i*_ and *ER* values represent a worse fit to the data, following the methodology of Anderson (2008). The best performing toxicity submodel for each case study is highlighted in bold.

	Helling et al. (2000)		Maboeta et al. (2004)		Zhou et al. (2007)		Zhou et al. (2011)	
Food availability	Optimal		Limited		Near optimal		Near optimal	
Sample size	59		30		30		12	
Toxicity submodel	Δ_*i*_	*ER*	Δ_*i*_	*ER*	Δ_*i*_	*ER*	Δ_*i*_	*ER*
T1	108.3	3.3 × 10^23^	122.6	4.1 × 10^26^	82.7	9.1 × 10^17^	30.7	4.6 × 10^6^
T2	87.4	9.5 × 10^18^	124.9	1.3 × 10^27^	70.8	2.4 × 10^15^	34	2.4 × 10^7^
T3	0	**1**	124.9	1.3 × 10^27^	0	**1**	0	**1**
T4	105.5	8.1 × 10^22^	0	**1**	77.3	6.1 × 10^16^	34.7	3.4 × 10^7^
