# Supplementary material for: An energy budget agent-based model of earthworm populations and its application to study the effects of pesticides
Source: Ecol Modell. 2014 May 24;280:5–17. doi: 10.1016/j.ecolmodel.2013.09.012 (PMC4375675; doi:10.1016/j.ecolmodel.2013.09.012)
Supplement: Supplementary file 1 [file mmc1.docx]

**Appendix A: TRACE Documentation**

This TRACE document was produced according to Schmolke et al. (2010). A revised version of this TRACE document, following the updated description of TRACE in Grimm et al. (“”; included in this special issue of Ecological Modelling), can be found in the supplementary material of Grimm et al.. “.

**1. Model Development**

*1.1. Problem Formulation*

The purpose of the model is to extrapolate from lower tier toxicity experiments for the OECD recommended earthworm species *Eisenia fetida*, in order to interpret the sublethal effects of toxicants at the field level. This forms a foundation for the application of the model in predicting pesticides (organics and inorganic) effects on earthworm populations under field conditions. We synthesise knowledge on the effects of pesticides on individual physiology and develop and evaluate methods for identifying the potential underlying physiological parameters toxicity disrupts. The broader model aim is to act as a potential refinement option in current risk assessments of soil invertebrates (SANCO, 2002; 2010). Thus, stakeholders include relevant agrochemical regulators and plant protection product (PPP) developers and risk assessors. Specific questions addressed by the model are: 1) Which physiological parameters are affected by toxic stress of different pesticides? 2) How does imposed stress on specific physiological parameters within the model translate into changes in growth, reproduction and survival rates? 3) How do variab­le background conditions (e.g. temperature, food availability) interact with toxic stress at the individual level? Environmental variables considered in the model are food density, soil temperature and soil moisture, which together determine the ecological niche of *E. fetida* (Reinecke & Viljoen, 1990; Tripathi & Bhardwaj, 2004; Edwards & Bater, 1992). As *E. fetida* is of importance in vermicomposting of animal wastes and is the recommended OECD test species (OECD, 1984), literature data is available on individual life cycle processes e.g. growth and reproduction and the variation of these rates under optimal to limiting environmental conditions. Model outputs are compared to literature data to assess the models applicability as a potential foundation for generating useful tools for predictive toxicology. Although extrapolations to the field level are possible when toxic stress is absent, literature data is not available for validating field applications of pesticides for this species. Thus, the model is currently applicable at the laboratory scale only, and outputs on growth and reproduction are evaluated over short periods of chemical exposure (< 56 days) and recorded at regular time-steps. Future applications of the model will be within an ecotoxicological framework, simulating field applications of pesticides on the population dynamics of ecologically relevant earthworm species.

*1.2. Design and Formulation*

The modelling approach used here combines energy budget and agent based modelling. Each individual in the model has its own energy budget, which includes algorithms for how energy uptake and expenditure direct life cycle processes. Individuals ingest food from their environment, assimilate a proportion of energy and expend available energy on maintenance, growth and reproduction. The destinations for allocated energy are listed in order of priority in Fig. A1. Maintenance is essential for the survival of an individual, and thus has first priority for energy allocation. Juveniles grow until sexually mature, and thereafter adults preferentially allocate energy to reproduction before growth. If energy remains after growth and/or reproduction, energy is stored in the energy reserves as glycogen (Byzova, 1977), which may be used to pay maintenance costs when food is limited. Energy budgets are important because the prioritisation of individuals will influence the population dynamics and demographics. This becomes of particular interest when studying toxic effects, as different physiological changes in individuals will result in different population level responses. Field populations are also exposed to many environmental stressors such as food limitation and low or high temperatures, which will result in physiological limitations which could amplify toxicity effects. For example, the population level effects of chemicals affecting the ingestion of food resources will be amplified in summer due to higher metabolic costs at high temperatures. Combining energy budget and agent based modelling approaches allows for interactions between individual energy budgets with stress exposure in mapped landscapes.

*
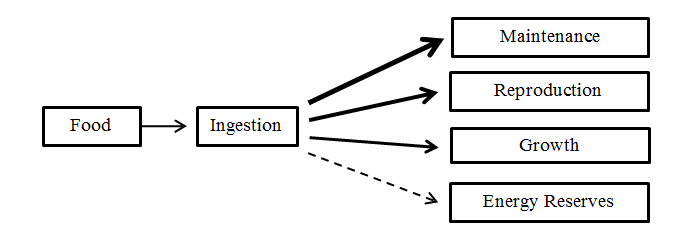
*

**Fig. A1.** Structure of the energy budget model for adults, with the thickness of arrows indicating priorities for energy allocation from food. Cocoons and juveniles are also in the model though cocoons do not grow and juveniles do not reproduce. Energy remaining after allocation enters the energy reserves which may be used for other functions when food is limited.

Key processes in the model determine how energy consumption and expenditure direct life cycle processes in response to environmental (e.g. food availability) and toxic stress. Individual energy budgets follow fundamental principles of physiological ecology (Sibly and Calow, 1986) and scale with body mass and temperature according to known allometric laws (Sibly et al., 2013). We aim to identify the physiological pathways of a toxicant by testing potential toxicity submodels which impose stress on different physiological parameters, the best performing submodel providing good fits to literature data on sublethal endpoints under different chemical concentrations. The energy budget model is incorporated within an agent based model (ABM) framework so that the biological responses of individuals to environmental and chemical stress can be realistically modelled. Of particular importance is the depletion of food resources by individuals. Entities within the model are individual *Eisenia fetida* and landscape patches. Interactions between individuals with their own energy budgets and landscapes comprising food density, temperature and pesticide concentrations result in density dependent regulation of populations, realistic of laboratory and field conditions. Although this approach is not as simple as others, such as matrix models, the simulated individual-landscape interactions are an important factor in extrapolating to the field scale.

*1.3. Model Description*

The model description follows the ODD protocol for describing ABMs (Grimm et al., 2010). The model is implemented in Netlogo 5.0.2 (Wilensky, 1999), a platform for building ABMs.

*Entities, state variables and scales*

This ABM comprises a number of individual *E. fetida* individuals and a model landscape consisting of two dimensional 0.01 m^2^ patches of soil. Individuals are characterized by life cycle stage (cocoon, juvenile or adult), mass and energy reserves, and landscape patches by pesticide concentration, food density, soil temperature and soil moisture. The model proceeds in discrete daily time-steps. Metabolic calculations are in units of energy per unit time (kJ day ^-1^).

*Process overview and scheduling*

The model proceeds in discrete daily time-steps, at the end of which individual and landscape state variables are updated. Juveniles and adults move randomly in the landscape, assimilating a fixed proportion of energy from ingested food that fuels life cycle processes and survival. Feeding by individuals depletes landscape patches and the food density changes accordingly. Cocoons cannot feed or move but pay maintenance costs from energy reserves until they are fully developed at the end of the temperature-dependent incubation period, when they hatch as juveniles (Sousa et al., 2008). Juveniles transform to adults once they reach a body mass threshold for sexual maturity (M­a, 1984; Springett and Gray, 1992). Food was provided in the same amounts as in the experiment being simulated, and food densities in landscape patches depleted as individuals ingested food. When food was not available, energy reserves were used to cover maintenance costs. Once the energy reserves are depleted to a critical level individuals catabolise energy from tissue to meet maintenance demands. Pesticides were applied in the ABM at the concentrations and times specified in the experiment being simulated. Individuals experiencing these concentrations were affected as indicated by potential ‘toxicity submodels’. Fig. 2 gives an overview of processes occurring at the adult stage in each time-step under different feeding conditions.

**
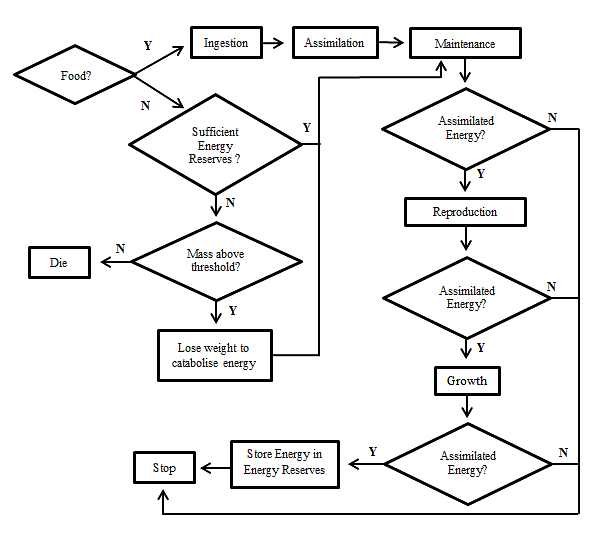
**

**Fig. A2.** Partial energy flow diagram of *E. fetida* adults within the ABM, showing the processes (rectangles) each individual goes through per time step, where diamonds express decision points. Energy reserves are used for maintenance and reproduction in starving individuals.

*Design Concepts*

*Emergence.* Variation in food availability between patches arises from the random movement and feeding of individuals across the landscape. Population dynamics emerge from differential energy allocation amongst individuals which is affected by pesticide concentration, soil temperature and food availability (Reinecke and Viljoen, 1990; Tripathi and Bhardwaj, 2004; Edwards and Bater, 1992).

*Interaction.* Individuals need a mate (any other adult as earthworms are hermaphrodite (Dominguez et al., 2003)) present in the same patch to reproduce. Adults and juveniles interact indirectly by competing for food within patches, and both affect patches by depleting food. Where food resources are limited individual ingestion rates are recalculated by dividing Ingestion Rate × Food Density by the sum of individual ingestion rates in the respective patch.

*Stochasticity.* Movement and background mortality are random amongst juveniles and adults, with specified probability density functions.

*Observation.* Population density, stage class structure (cocoon, juvenile, adult), individual reproduction and growth rates were analysed.

*Initialization*

Simulations were initialised with individuals randomly distributed in the landscape. Landscape size, soil temperature and moisture, food density, earthworm numbers, life cycle stages and body masses followed the experiment being replicated, outlined in detail in section *2.3.1* and *3.1.1*. Species-specific parameters were derived from the literature for *E. fetida* as shown in Table 1. Where data were not available for *E. fetida* closely related species were used. For example, the assimilation efficiency estimated by Hobbelen & van Gestel (2007) was for *Lumbricus rubellus*, which we suggest is similar for *E. fetida* given its epigeic feeding strategy. Where parameter values were not available calculations were necessary, as documented in *1.4. Parameterisation.*

| **Table A1.** Default parameter values for the energy budget model with reference to literature data sources | | | | | |
| --- | --- | --- | --- | --- | --- |
| **Symbol** | **Definition** | **Value** | **Unit** | **Reference** | **Notes** |
| *A_e_* | Assimilation efficiency | 0.50 | --- | Hobbelen and van Gestel (2007) | p. 376 |
| *B_o_* | Taxon-specific normalization constant | 967 | kJ/day | Meehan (2006) | Calculated from Table 2, p. 881 and Eq. 4 |
| *E* | Activation energy | 0.25 | eV | Meehan (2006) | p. 880 |
| *E_c_* | Energy content of tissue | 7 | kJ/g | Peters (1983) | p. 235 |
| *E_s_* | Energy cost of synthesis | 3.6 | kJ/g | Sibly and Calow (1986) | Calculated from p. 54-55 |
| *E_x_* | Energy content of food | 21.2 | kJ/g | Wang et al. (2011) | p. 173 |
| *IG_max_* | Maximum ingestion rate | 0.70 | g/(day/g) | Neuhauser et al. (1980) | Derived/re-calculated from Fig. 6, p. 96 |
| *h* | Half saturation coefficient | 3.5 | g/unit area | Neuhauser et al. (1980) |  |
| *M_b_* | Mass at birth | 0.011 | g | Gunadi et al. (2002) | Derived from Table 1, p. 18 and Fig. 1, p. 19 |
| *M_c_* | Mass of cocoon | 0.015 | g | Hartenstein et al. (1979) | Derived mean from Fig. 5, p. 333 |
| *M_p_* | Mass at sexual maturity | 0.25 | g | Gunadi et al. (2002) | Derived from Table 1, p. 18 and Fig. 1, p. 19 |
| *M_m_* | Maximum asymptotic mass | 0.50 | g | Gunadi et al. (2002) |  |
| *r_B_* | Growth constant | 0.177 | --- | Gunadi et al. (2002) | Fig. 1, p. 19 fitted to Eq. 5a |
| *r_m_* | Maximum rate of energy allocation to reproduction | 0.182 | kJ/g  day | Tripathi and Bhardwaj (2004) | Derived from p. 281 |
| *T_0_* | Incubation period | 23 | Days | Reinecke et al. (1992) | Table 3, p. 1298 |
| *T_ref_* | Reference temperature | 298.15 | K | Tripathi and Bhardwaj (2004) | p. 280 |

*Submodels*

The following sections describe the energy budget model, outlined in the above sections and in brief in Fig. A1, in terms of metabolic organisation at the individual level.

*Maintenance*

The basal metabolic rate (*B*) is the level of metabolism below which an organism cannot survive (Fry, 1971; Calow & Sibly, 1990), and is used here as a measure of maintenance costs. Costs of movement, small in earthworms, are here included in maintenance. *B* is known to scale with body mass (*M*) as a power law and temperature (*T*), measured in grams and kelvins respectively, according to the equation:

${B=B_{0}M^{3/4}e^{{-E}/{kT}}}$ Eq. A1

where *B*_o_ is a taxon-specific normalization constant, *M^3/4^* is the scaling with body mass, *e^-E/κT^* is the exponential Arrhenius function, *E* is the activation energy, *κ* is the Boltzmann’s constant (8.62 x 10^-5^ eV K^-1^) and T_ref_ is a reference temperature (Table A1) (Peters, 1983; Gillooly et al., 2001; Brown & Sibly, 2012). The effect of temperature on metabolic rate, $e^{\frac{-E}{\kappa} \left( \frac{1}{T}-\frac{1}{T_{ref}} \right)}$,$e^{\frac{-E}{\kappa} \left( \frac{1}{T}-\frac{1}{T_{\mathrm{ref}}} \right)}$ is referred to as the Arrhenius function.

*Ingestion and Energy Uptake*

Variation in food density affects the rate of ingestion of food up to an asymptote according to a type II functional response (Holling, 1959; Ricklefs & Miller, 2000), so that:

Ingestion rate $\propto\frac{X}{(h+X)}$

where X is food density and *h* is a constant that shows how quickly the response curve reaches its maximum as food density increases. The ingestion rate is proportional to the surface area (*M*^2/3^) of an individual as the search rate depends on the food gathering apparatus (Kooijman & Metz, 1984, Pilarska, 1977) and also on the effects of body mass and temperature, giving:

Ingestion rate $= {IG}_{max} e^{\frac{-E}{\kappa} \left( \frac{1}{T}-\frac{1}{T_{ref}} \right)}\frac{X}{(h+X)} M^{2/3}$ Eq. A2

where *IG*_max_ is the maximum ingestion rate recorded for *E. fetida* under optimal feeding conditions (g/(day/g)). After ingestion, food is processed by the digestive system and a proportion, governed by the energy content of food and assimilation efficiency, becomes available for allocation to the various functions shown in Fig. 1. The value of the assimilation efficiency (*A*_e_) (Table A1) depends on diet but not body mass (Hendriks, 1999).

*Growth*

After expenditure to maintenance and, in the adult stage, to reproduction, individuals allocate remaining energy to somatic growth. The maximum growth rate of an individual under optimal conditions is assumed to follow the von Bertalanffy (1957) growth equation:

${M=M_{m}(1-(1-\left( \frac{M_{b}}{M_{m}} \right)}^{1/3}) e^{-r_{B}t/3})^{3}$ Eq. A3a

where *M*_b_ and *M*_m_ denote mass at birth and maximum mass respectively and $r_{B}$ is the Bertalanffy growth constant, obtained by fitting Eq. 3a to data recording the increase in individual biomass over time under optimal conditions. The maximum growth rate per time-step is obtained from:

$\Delta M= r_{B}e^{\frac{-E}{\kappa} \left( \frac{1}{T}-\frac{1}{T_{ref}} \right)}(M_{m}^{1/3} M^{2/3}-M)$ Eq. A3b

(Sibly et al, 2013). The energy costs of growth are determined from the new mass calculated from Eq. 3b and the energy costs of production (*E*_c_ + *E*_s_)$(E_{c}+E_{s})$ (Table A1). Eq. 3b shows how the maximum rate at which resources can be allocated to growth changes as an individual increases in mass. If insufficient energy is available to support maximal growth, growth rate is reduced accordingly.

*Reproduction*

Reproduction is assumed to take priority over growth in adults, because in the absence of a sexual partner, indeterminate growers grow larger than normal (West et al., 2001). Energy allocated to reproduction by adults goes directly to the production of an egg until oviposition inside a cocoon. The maximum rate of energy allocation to reproduction per day increases linearly with adult mass (Mulder et al., 2007):

$\Delta R= r_{m}e^{\frac{-E}{\kappa} \left( \frac{1}{T}-\frac{1}{T_{ref}} \right)}M$ Eq. A4

where *r*_m_ is the maximum rate of energy allocation to reproduction per unit of adult mass (kJ/g/day). The energy costs of producing a hatchling is *M*_c_(*E*_c_ + *E*_s_) (Table A1) and the hatchling’s energy content is initially *M*_c_.*E*_c_, , which is utilized for maintenance during the incubation period, based on Eq. 1 and *M*_b_.

*Energy Reserves and Starvation*

If any assimilated energy remains after expenditure on relevant life processes (Fig. 1) it is stored in an individual’s energy reserves. Energy is stored as glycogen (Byzova, 1977), costing *E*_s_ = 3.6 kJ to store 1g with an energy content of *E*_c_ = 7 kJ (Sibly & Calow, 1986; Peters, 1983). When energy is not available from ingested material, maintenance costs are taken from energy reserves, allowing individuals to survive for some time under starvation before dying (Sousa et al, 2008; Gunadi et al., 2002). Furthermore, as evidence supports the assumption that reproduction continues even when food is limiting (Reinecke and Viljoen, 1990), the energy reserves are assumed to be utilized for reproduction above a threshold of 50% of an individual’s maximum energy reserves, taken as $\frac{M}{2}E_{c}$ (e.g. Peters, 1983). If food limitation continues and the energy reserves decline to a critical level below 50% of an individual’s maximum energy reserves, individuals are considered to be in a state of starvation. Under these conditions tissue is catabolised to cover maintenance costs, resulting in net weight loss (Gunadi & Edwards, 2003); individuals die if their mass falls to that at birth (*M*_b_) following Reinecke & Viljoen (1990).

*Movement*

On the basis that Kobetičová et al. (2010) found movement in *E. fetida* individuals to be random, we modelled individual movements as random in direction from a uniform distribution between -90 ^o^ and 90 º and distance travelled as 0.5 patches per time-step.

*Survival*

The survival of individuals living in field populations is determined by the availability of energy resources to maintain life cycle processes alongside temperature and soil moisture specific mortality rates. Individuals die of starvation if their energy resources are depleted, and additional mortality rates were imposed using the regression equation derived from Presley et al. (1996):

Mortality Rate (%) = 12.7 – 0.0010 *SM* – 0.0861 *T* + 0.000009 *SM*^2^ + 0.000147 *T*^2^ $2531-0.20 SM-17.2 T+0.000187 \mathrm{SM}^{2}+0.00294 T^{2}$ Eq. A5

where *SM* is soil moisture (%) and *T* is soil temperature (K). Individual adults and juveniles die according to Bernoulli processes with daily mortality rates given by Eq. A5.

*Pesticide Applications*

The model is designed to simulate laboratory based toxicology experiments from the literature, typical of lower tier risk assessment*.* Pesticide applications are simulated by applying a chemical concentration to each patch at the specified concentration and time/s. Individuals experience the patch concentration on contact and the effect of this concentration persists unchanged for the duration of the experiment. The physiological effects of a pesticide were identified using toxicity submodels (Table A2), which were evaluated using data from the experiment being simulated. We converted individual biomass and cocoon production values during different treatment concentrations in each case study to percentages of the control value, to identify the reduction in sublethal endpoint due to chemical exposure. The data was then generally well fitted by exponentially declining curves, of the form:

$R(C){= e}^{(kC)}$ Eq. A6

where *R(C)* is the effect at a specific concentration (*C*) recorded as % compared to control, *k* is a chemical-specific coefficient calculated by regressing log (%trait compared to control/100) against chemical concentration (*C*) in mg/kg. Eq. 6 represents the dose-response relationship between chemical concentration and a life cycle trait (growth or reproduction), presented in Fig. 3. However, the toxicity data does not specify which physiological parameter was affected by exposure to result in the observed response in that life cycle trait and as the type of physiological response impacts the population’s response, we wanted to identify this.





**Fig. A3.** Modelling dose-response curves. Curves fitted to experimental laboratory data for a, c, e & g: growth and b, d & f: reproduction, for a & b: copper oxychloride by Helling et al. (2000); c & d: chlorpyrifos by Zhou et al. (2007), e & f: chlorpyrifos by Zhou et al. (2011) and g: copper oxychloride by Maboeta et al. (2004). R^2^ values for regression curves in a, b, c, d, e, f & g are: 0.81, 0.73, 0.65, 0.99, 0.92, 0.96 and 0.99 respectively. Reproduction and growth data are represented as a reduction in life cycle trait compared to the control under different concentrations. Regression coefficients determining these curves are used to investigate the putative metabolic pathway for each pesticide.

To find the most likely physiological parameter affected in each case study we investigated the various possibilities, here called toxicity submodels*.* Inspection of Fig. A1 indicates that chemicals can affect ingestion, assimilation, maintenance, growth or reproduction, the rates of which are governed by physiological parameters ${IG}_{max}, A_{e}, B_{0}, r_{B}$ or $r_{m}$ respectively (Table A1). Here, we investigate four potential toxicity submodels, describing how altering specific physiological parameters modifies individual growth and reproduction rates (Table A2). The four submodels tested here were selected on the basis that modifying the specific parameters has effects on growth and reproduction simultaneously, rather than one metabolic rate alone. This was done by supposing that the chemical-specific toxicity coefficient (*k*) obtained by fitting Eq. 6 to the data shown in Fig. A3 determines the relationship of the chemical concentrations with a physiological parameter, rather than with the life cycle trait, calculated as:

$P_{c}= \frac{P_{0}}{100}e^{(kC)}$ Eq. A7

where $P_{c}$ is the parameter value at concentration (*C*), $P_{0}$ is the parameter value under control conditions as indicated in Table A1 and *k* is the toxicity coefficient determining the dose-response relationship. Effects on the sublethal endpoints growth and reproduction then emerge from model simulations. For example, a decline in the value of the parameter ${IG}_{max}$ with increasing chemical concentration would reduce individual ingestion, thus reducing the amount of energy available for allocation to metabolic processes. Following the preferential allocation principles for earthworms this would lead to reduced growth but have little impact on reproduction as adults allocate energy preferentially to reproduction before growth*.* Toxicity submodel T4 requires an increase in the value of the maintenance parameter $B_{0}$ to eliminate/detoxify the toxin or repair damage (rather than a decline as in toxicity submodels T1-T3 which follow the dose-response curves in Fig A3). Here we assumed that above a concentration of 100 mg/kg there is a linear relationship between $B_{0}$ and *C* so that:

$B_{0}=B_{0 control}$, if *C* $\leq$ 100;

$B_{0}=B_{0 control} \times0.01 C$, if *C* > 100.

| **Table A2.** Tested toxicity submodels used to identify the physiological pathways disrupted by pesticides. In each case the specified physiological parameters were affected according to dose-response curves parameterised as in Fig A3. ${IG}_{max}$is maximum ingestion rate, $r_{m}$ is maximum rate of energy allocation to reproduction, $r_{B}$ is the von Bertalanffy growth constant and $B_{0}$ is a taxon-specific normalization constant used for calculating maintenance rates. | | |
| --- | --- | --- |
| **Toxicity Submodel** | **Parameter** | **Predicted Observations in Adult Life Cycle Traits** |
| T1 | ${IG}_{max}$ | Growth more reduced than reproduction |
| T2 | ${IG}_{max} \& r_{m}$ | Growth and reproduction similarly reduced |
| T3 | $r_{m} \&$ $r_{B}$ | Reproduction more reduced than growth |
| T4 | $B_{0}$ | Growth more reduced than reproduction or accelerated weight loss under resource limitation |

*1.4. Parameterization*

Where parameter values have not been directly taken from the literature data source in Table A1, calculations were necessary to obtain a best estimate.

*1.4.1. Assimilation efficiency,* $A_{e}$

Assimilation efficiency determines how much energy from the ingested food (determined by *IG_max_* and E_X_; Table A1) becomes available for expenditure to metabolic processes (Fig. A1). As *E. fetida* feeds on resources high in organic matter, the assimilation efficiency is assumed to be relatively high. Here we follow Hobellen and van Gestel (2008) who used a value of $A_{e}$ = 50% for the epigeic species *L. rubellus.* This is less than the value of 70% recorded for *L. rubellus* eating alder leaves by Dickschen and Topp (1987), but we consider it realistic as the energy content of cow manure, a common food of *E. fetida* in the conditions simulated here, is much higher than that of plant material.

*1.4.2. Normalization constant for maintenance, B_0_*

Regression analysis of earthworm data by Meehan (2006 p. 881) yielded

$\ln\left( B \right)= 5.70+0.71\ln\left( M \right)-0.25/\kappa T$ Eq. A8

in the notation of Eq. A1, where *B* is measured in J/hour and *M* in mg. Evaluated at M = 1g and T = 298.15 K (25 °C) gives *B* = 0.0577 kJ/day, which can be used to yield a B_0_ value of 967 kJ/day.

*1.4.3. Ingestion Rate, IG_max_*

The maximum ingestion rate (IG_max_) was calculated from growth data of individual *E. fetida* recorded by Neuhauser et al. (1980) (Fig. 6, p. 96) under varying densities of cow manure. Calculations are shown in Table A3. Earthworms continued to growth for 8 weeks at each food ration, and so it is assumed that ingestion occurred until day 56. Thus, the mean amount of food ingested (g) per day at each food ration is calculated as original food ration (g)/56 days (column 2, Table A3). The mean biomass (g) of individuals (column 3) was taken from Fig. 6 of Neuhauser et al. (1980). Individual ingestion rates were re-calculated for 1g individuals (column 4, Table A3), as all metabolic rates scale with mass, which gives a value for IG_max_ of 0.70 g/(day/g) (Table A1).

| **Table A3.** Calculations for the estimation of maximum ingestion rate (IG_max_) in g/day for *Eisenia fetida*. Derived from Neuhauser et al., 1980. | | | |
| --- | --- | --- | --- |
| **Food Ration (g)** | **Ingested food (g/day)** | **Mean individual biomass (g)** | **Ingestion rate (g/(day/g))** |
| 5 | 0.089 | 0.21 | 0.42 |
| 10 | 0.179 | 0.30 | 0.60 |
| 15 | 0.268 | 0.42 | 0.64 |
| 20 | 0.357 | 0.51 | 0.70 |

*1.4.4. Half Saturation Coefficient, K*

The maximum ingestion rate (*IG*_max_) was calculated from growth data of individual *E. fetida* recorded by Neuhauser et al. (1980) (Table A3). The data follow a Holling Type II response curve in which the value of the half saturation coefficient (*h*) corresponds to the food ration at which ingestion is half its maximum, here calculated as 3.5 g (Fig. A4).


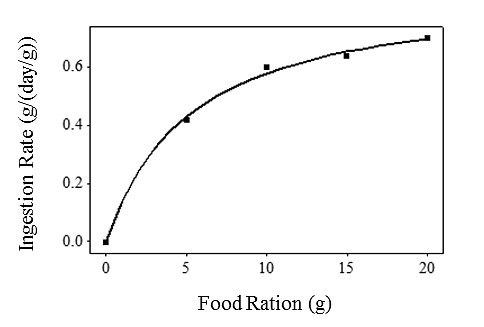


**Fig. A4.** Ingestion rates for *E. fetida* under varying food densities (derived from Neuhauser et al., 1980), with the fitted curve representing the Holling Type II functional response.

*1.4.5. Growth Constant,* $r_{B}$

The growth constant was obtained by least-squares regression fit of the von Bertalanffy growth equation (Eq. 4a) to data from Gunadi et al. (2002) at 20 °C, giving *r_B_* = 0.15 (Fig. A5). The Arrhenius function was used to correct this to 0.177 at 25 °C, considered the optimal temperature for development of *E. fetida* (Tripathi and Bhardwaj, 2004).


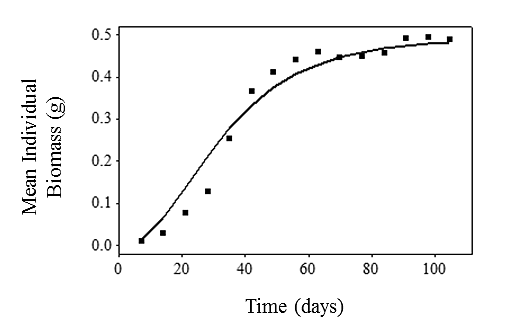


**Fig. A5.** von Bertalanffy growth curve (line) fitted to growth data from Gunadi et al. (2002) (points), yielding a value for rB of 0.15 at 20 °C (R^2^ = 0.97).

*1.4.6. Maximum rate of allocation to reproduction, r_m_*

Tripathi and Bhardwaj (2004) (p. 281) recorded a maximum cocoon production of 4.4 per individual per month with an average 3 hatchlings emerging from each cocoon. This gives a reproduction rate of 13.2 hatchlings per individual per month and 0.44 hatchlings individual^-1^ day^-1^. Taking the energy costs of producing one cocoon as $M_{c}{(E}_{c}+E_{s})=0.159 \mathrm{kJ}$, this gives a value for energy allocated to reproduction by an average individual as 0.07 kJ day^-1^. Taking 0.385 g as the average adult mass gives r_m_ = 0.182 kJ/g day^-1^.

**2. Model Testing and Analysis**

*2.1. Verification*

The model was thoroughly tested to verify that the model behaves as expected. Initial model testing focused on the underlying components of the energy budget model on which individual life histories depend. Alongside checking metabolic rates under different conditions against Excel spread-sheets, we altered components that produce predictable changes in output. For example, if the reproduction submodel is removed individuals grow to a larger size. More general testing used information from the literature on mortality at different temperatures, life-spans, maximum reproduction rates and carrying capacities of field populations, to verify overall model performance. Major tests conducted due to problems occurring during model development are outlined below. The model code was further independently checked for bugs.

*Growth*

Under parameterisation (*1.4.*) the growth constant parameter was taken as 0.15 without the consideration of temperature effects on this value. Thus, when growth simulations were conducted for verification against literature data recorded at 25 °C growth was under-predicted. The growth constant was converted to 0.177 under 25 °C using the Arrhenius function, which when tested against growth under 20 °C (Gunadi et al., 2002) and 25 °C (Reinecke and Viljoen, 1990) produced a better fit to the data.

*Reproduction*

Initially, cocoons were assumed to contain 3 ova with an energy content of $3(M_{c}{(E}_{c}+E_{s}))$. Adults thus had to obtain the associated amount of energy to reproduce. Yet cocoon production under variable feeding conditions suggests that once food becomes a limiting factor adults produce cocoons containing only 1 ovum. Further model testing against the cocoon production experiments conducted by Reinecke and Viljoen (1990) under variable food densities showed that depositing cocoons with single ova gave a better fit to the data.

*Starvation*

Little information exists on the metabolism of earthworms under starvation. Thus, it was necessary to use the available data to determine relevant metabolic algorithms. Initially, the loss of mass under starvation was assumed to follow an inverse relationship to the growth equation (Eq. 3b). Yet, closer inspection and testing against weight loss data from Gunadi et al. (2002) showed this to be over-predicted. As maintenance and specifically basal metabolic rate (BMR) is said to be essential for survival under limiting food conditions, weight loss was assumed to be proportional to the energy costs of BMR.

*2.2. Local Sensitivity Analysis*

The sensitivity of the model to the values of its parameters is presented in Table A4. The model was run with the parameter values of Table A1 (N=100) and again with parameter values increased one at a time by 10% (N=100) and changes in model outputs (adult biomass, juvenile biomass and cocoon production per adult) are shown in Table A4. Also shown in Table A4 are the sensitivity of the model to the baseline values of the environmental variables varied individually; these were soil temperature: 25 °C; soil moisture: 60%; and food density: 20g per patch. All simulations were run for one year under the field study conditions outlined in Section *2.3.2*. Sensitivity analysis shows the model to be generally robust to changes in parameter values (sensitivities < 1), with only activation energy (*E*) and reference temperature (*T_ref_*) yielding ratios >1 (Table A4). An increase in activation energy reduces maintenance costs, making more energy available for higher growth and reproduction rates, whilst an increase in the reference temperature alters the Arrhenius function. All environmental variable values yielded sensitivities in the range -1 to +1.

| **Table A4.** Sensitivity analysis showing ratio of % changes in mean output variables to 10% changes in parameter values, with standard errors. Thus sensitivities between -1 and +1 represent changes in outputs between -10% and +10% of baseline values respectively. | | | |
| --- | --- | --- | --- |
|  | **Output Variables** | | |
| **Parameter** | **Adult Biomass** | **Juvenile Biomass** | **Cocoons per Adult** |
| Assimilation efficiency (*A_e_*) $(A_{e})$ | 0.03 ±0.08 | 0.02 ± 0.14 | 0.04 ±0.09 |
| Taxon-specific normalization constant (*B_o_*)$B_{0})$ | 0.12 ±0.10 | 0.09 ±0.08 | 0.18 ±0.14 |
| Activation energy (*E*) | 1.09 ±0.31 | 1.18 ±0.19 | 1.17 ±1.11 |
| Energy content of tissue (*E_c_*)$E_{c})$ | 0.12 ±0.11 | -0.41 ±0.19 | 0.04 ±0.12 |
| Energy cost of synthesis (*E_s_*)$E_{s})$ | 0.13 ±0.11 | -0.01 ±0.03 | -0.06 ±0.12 |
| Energy content of food (*E_X_*)$E_{X})$ | -0.06 ±0.04 | 0.08 ±0.07 | 0.11 ±0.08 |
| Maximum ingestion rate (*IG_max_*)$\mathrm{IG}_{\max})$ | -0.26 ±0.09 | 0.01 ±0.09 | -0.10 ±0.11 |
| Half saturation coefficient (*h*) | 0.01 ±0.02 | 0.01 ±0.06 | 0.03 ±0.05 |
| Mass at birth (*M_b_*)$M_{b})$ | -0.02 ±0.06 | -0.01 ±0.16 | -0.10 ±0.04 |
| Mass at sexual maturity (*M_p_*)$M_{p})$ | 0.07 ±0.09 | 0.26 ±0.11 | 0.01 ±0.09 |
| Maximum asymptotic weight (*M_m_*)$M_{m})$ | 0.03 ±0.07 | 0.35 ±0.12 | -0.05 ±0.10 |
| Mass of cocoon (*M_c_*)$M_{c})$ | 0.19 ±0.13 | -0.08 ±0.01 | -0.10 ±0.18 |
| Growth constant (*r_B_*)$r_{B})$ | -0.02 ±0.10 | 0.08 ±0.12 | 0.08 ±0.10 |
| Maximum rate of energy allocation to reproduction (*r_m_*)$r_{m})$ | 0.02 ±0.07 | 0.01 ±0.09 | 0.03 ±0.01 |
| Incubation period (*T_0_*) | 0.02 ±0.03 | 0.01 ±0.10 | -0.02 ±0.08 |
| Reference temperature (*T_ref_*) | 1.42 ±0.13 | -0.95 ±0.13 | 1.03 ±1.04 |
| Environmental Variable |  |  |  |
| Soil Temperature (*T*) | 0.48 ±0.15 | -0.52 ±0.09 | 0.25 ±0.22 |
| Soil Moisture (*SM*) | 0.01 ±0.10 | 0.01 ±0.08 | 0.02 ±0.01 |
| Food Density (*X*) | -0.17 ±0.08 | 0.13 ±0.09 | -0.09 ±0.03 |

*2.3 Validation*

The performance of the model is evaluated by comparing its outputs with the results of laboratory and field studies, using the approach of pattern-oriented modelling (POM) (Grimm et al., 2005).

*2.3.1. Laboratory Experiments*

The model was set up to simulate the conditions of a number of published laboratory experiments to evaluate model fits to growth and reproduction data, outlined in Table A5. The studies of Gunadi et al. (2002), Gunadi & Edwards (2003) and Reinecke & Viljoen (1990) were used to evaluate model fits to growth and reproduction under variable food availability and temperature. Food quantities were uniformly distributed over the landscape at the feeding times indicated in Table 2. Gunadi and Edwards (2003) recorded a mortality rate of 28 % before 161 days and added 10 of the surviving adults to a new substrate on day 161, which is simulated in the model as shown in Table A5. No mortality occurred in the other simulated laboratory experiments.

| **Table A5.** Experimental conditions used in model simulations for comparison with growth and reproduction data from the cited studies. Soil moisture content was 80% in all experiments. | | | | | |
| --- | --- | --- | --- | --- | --- |
| **Study** | **Fig.** | **Number of Individuals** | **Food Quantity (g)** | **Feeding Times**  **(days)** | **Temperature (ºC)** |
| Gunadi et al. (2002) | A6a. | 5 | 150 | 0 | 20 |
| Gunadi & Edwards (2003) | A6b. | 8, 6/5, 10 | 100 | 0, 161 & 315 | 20 |
| Reinecke & Viljoen (1990)  Reinecke & Viljoen (1990)  Reinecke & Viljoen (1990) | A6c. | 10 | 10, 50 & 25 | 10, 60 &140 | 25 |
|  | A7a. | 10 | 20 | per 20 days | 25 |
|  | A7b. | 10 | 10, 50 & 25 | 10, 60 & 140 | 25 |

Comparisons of model outputs with literature data on growth and reproduction, under the conditions, are presented in Figs. A6 & A7. Simulation of Gunadi et al.’s (2002) experiment shows a good match to data in both the increasing phase (optimal food) and the descending phase when individuals lost body mass because the food supply was depleted (Fig. A6a). Model predictions of mass loss during starvation are less accurate in Figures A6b and c, but the discrepancies are in opposite directions, so it would be hard to fit both datasets well.





**Fig. A6.** Comparison between model outputs (lines) and recorded growth data (points) from a) Gunadi et al. (2002) b) Gunadi & Edwards (2003) and c) Reinecke & Viljoen (1990). Arrows indicate the time and amounts of food supplied.

Reinecke & Viljoen (1990) recorded the reproduction rate of *E. fetida* under optimal (20g cow manure every 10 days) and limiting conditions (Fig. A7). Model outputs for reproduction under both optimal and limiting food conditions fit the experimental data well.


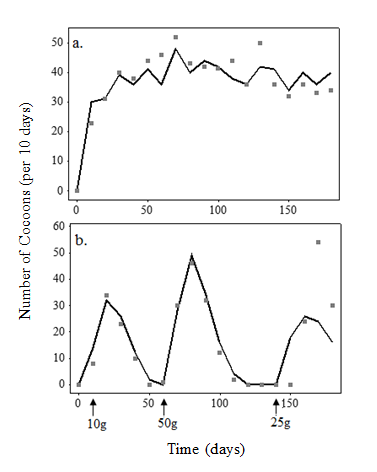


**Fig. A7.** Comparison between reproduction data (number of cocoons produced by 10 individuals in 10-day intervals) from the a) control group with optimal feeding conditions (20g cattle manure every 20 days) and b) experimental group with limiting food conditions in Reinecke & Viljoen (1990) (points) and model simulations (lines). Arrows indicate the time and mass of food supplied.

*2.3.2. Population dynamics in the field*

The model was used to simulate *E. fetida* population dynamics under the field conditions studied by Monroy et al. (2006) (Fig. A8). The authors collected data from a manure heap exposed to seasonal environmental conditions over one year. Autumn, winter, spring and summer rainfall (mm)/ambient temperature (°C) were recorded as 600/9.1, 200/8.1, 770/12.1 and 250/16 respectively, but the detailed environmental data needed for our model were not reported and therefore had to be estimated as follows. We followed Meyer’s (1926) suggestion that soil moisture content is equivalent to precipitation (mm) divided by the saturation deficit (mm Hg) of air, and assumed an average annual humidity of 50%, giving a saturation deficit of 20 mmHg (Frankham, Ballou & Briscoe, 2004). Manure was assumed to have a 30% higher water holding capacity than soil, following Unger & Stewart (1974). Petersen, Lind & Sommer (1998) found large cattle manure heaps to be 2.3 – 6.2 °C warmer than the surrounding air at a depth of 50cm, with a mean air temperature of 10 °C, and on this basis we assumed a compositional warming effect of 5 °C for ambient temperatures reported over 10 °C as the manure heap in Monroy et al. (2006) is described as “a temporary heap of cow manure from a small farm”. These calculations give soil moisture (%)/soil temperature (°C) values of 60/9, 40/8, 70/17 and 45/21 for autumn, winter, spring and summer respectively. As food density was not measured in the original study estimates of seasonal availability were made as follows. Maximum quantities of 350 kg manure/heap were reported by Rufino et al. (2007) for large dairy farms. As the manure heap in Monroy et al. (2006) was only a temporary heap on a small farm measured per m^2^ rather than per heap, the maximum quantity of manure was taken as 50 kg / m^2^. Seasonal variation was estimated on the basis that more cattle feed is provided during winter and spring, that decomposition rates increase in summer, and that most of the manure heap was removed in late spring each year (Monroy et al., 2006), yielding values of 10, 15, 50 and 5 kg / m^2^ of manure for autumn, winter, spring and summer respectively. Although these are mere approximations they are the best estimates available. Soil temperature and moisture values were assumed to vary according to a normal distribution with SD 5 ºC and 10% respectively. Food densities were randomly distributed across the 1m^2^ landscape patches to account for spatial heterogeneity, taken from a normal distribution with SD 10% of the mean.


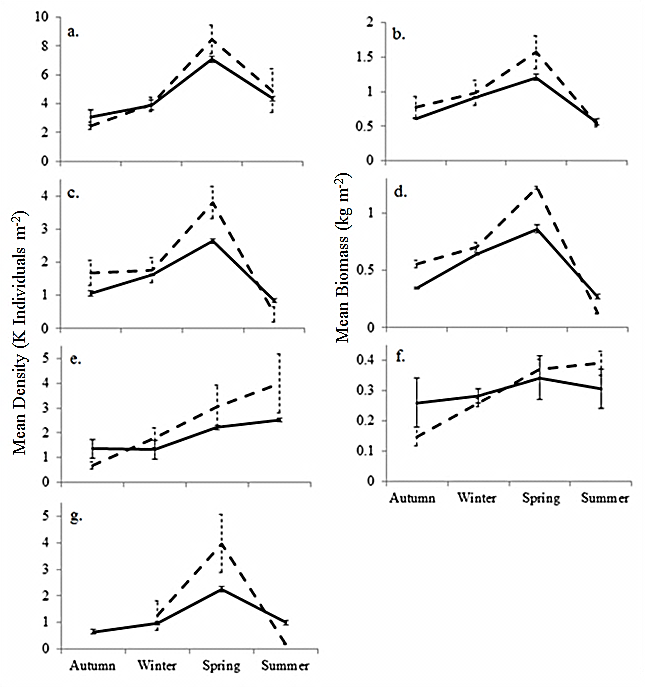


**Fig. A8.** Comparison between (left-hand panels) field population density data and (right-hand panels) population biomass data from Monroy et al. (2006) (dashed line) and model simulations (solid lines) with standard errors. a,b: total population; c,d: adults; e,f: juveniles; g: cocoons. Juveniles here comprise the hatchlings, juveniles and preclittelates that were counted separately in the field.

Patterns of seasonal changes in population density and biomass (Figs A8a and b) are generally well predicted by the model, although adult density and biomass and cocoon density are slightly underestimated in spring. The higher cocoon densities observed in spring may be due to higher temperatures occurring within the manure heap under high population densities, not considered in the model. Overall, the model captures the major aspects of the population dynamics recorded by Monroy et al. (2006) but would have benefited from more detailed recordings of abiotic factors such as soil temperature and organic matter content during the field study.

*2.3.3. Validation Methods*

*Pattern Oriented Modelling*

Model outputs are compared to experimental data from the literature on both individual life cycle processes and population dynamics using pattern-oriented modelling (POM) (Grimm et al., 2005). POM states that if a model can approximate the real system it attempts to predict it can be considered robust, and assumed to capture the underlying mechanisms which drive the system. Thus, good model fits to individual life cycle and field population data in the absence of toxic stress (Figs. A6 – A8) validate the models ability to predict earthworm physiology under differing environmental conditions. Discrepancies arising between the model and data may be explained by differences in experimental conditions. For example, the over-prediction of maximum body mass in Fig. A6b may be related to food quality since the cow manure in this experiment was pre-composted, known to result in reduced earthworm growth when used as a food source (Gunadi et al., 2002). An underestimate of mass loss during starvation in Fig. A6c may similarly relate to the provision of more energy-rich food resources, leading to an overestimate of the amount of food digested and therefore energy assimilated. These discrepancies highlight the need for comprehensive environmental measurements in experimental work. Conversely, some discrepancies may be a product of assumptions not being considered, in an attempt not to over-complicate the model. For example, an under-prediction of cocoon density in spring (Fig. A8g.) may have been due to increasing temperatures of the manure heap under high population densities, not considered by the model.

*Goodness of Fit Tests*

As a supplement to POM, we evaluate the fits of the model to the data using simple goodness of fit tests. The adequacy of model parameterisation was evaluated by comparing predictions from the model when its parameters took the values in Table A1, to predictions produced when parameters took random values. Goodness of fit was calculated as the sum of squared differences between model predictions and the data shown in Figs A6 – A8. To make the evaluation as rigorous as possible, only relevant parameters were randomised: these were $E_{c}, E_{s}, M_{p}, M_{m}, M_{c}, r_{m}$ for growth (Fig. A6), $E_{X}, {IG}_{max}, M_{b}, M_{p}, M_{m}, r_{B}$ for reproduction (Fig. A7), and all parameters for population dynamics (Fig. A8). Table A6 shows that predictions using Table A1 values performed better (p < 0.05) than predictions using randomised parameter values in each of 12 independent tests using the data described in sections 2.3.1 and 2.3.2. This supports the model parameterisation shown in Table A1 and section *1.4.*

| **Table A6.** Results of randomisation tests evaluating the adequacy of model parameters. In each randomisation parameters were chosen randomly from a uniform distribution ranging from zero to twice the value in Table A1. 100 replicates are used for high frequency outputs (e.g. population density) whilst 50 replicates are used for lower frequency outputs (e.g. individual biomass). *n =* number of randomisations; p = proportion of randomly parameterised models that fit the data better than those produced using the justified parameters presented in Table A1. | | | |
| --- | --- | --- | --- |
| **Study** | **Fig.** | ***N*** | ***P*** |
| Gunadi et al. (2002) | A6a. | 50 | 0.00 |
| Gunadi & Edwards (2003) | A6b. | 50 | 0.00 |
| Reinecke & Viljoen (1990) | A6c. | 50 | 0.02 |
| Reinecke & Viljoen (1990) | A7a. | 50 | 0.00 |
| Reinecke & Viljoen (1990) | A7b. | 50 | 0.00 |
| Monroy et al. (2006) | A8a. | 100 | 0.00 |
| Monroy et al. (2006) | A8b. | 100 | 0.00 |
| Monroy et al. (2006) | A8c. | 100 | 0.02 |
| Monroy et al. (2006) | A8d. | 100 | 0.00 |
| Monroy et al. (2006) | A8e. | 50 | 0.00 |
| Monroy et al. (2006) | A8f. | 50 | 0.00 |
| Monroy et al. (2006) | A8g. | 50 | 0.00 |

**3. Model Application**

*3.1. Results*

*3.1.1. Model Simulations*

The model was set up to simulate the conditions of a number of laboratory experiments to evaluate model fits to growth and reproduction data, using the best performing toxicity submodel. Identification of the best performing toxicity submodel is described in the next section (3.1.2). The studies of Helling et al. (2000) and Maboeta et al. (2004) were used to investigate the effects of copper oxychloride, those of Zhou et al. (2007) and (2011) for chlorpyrifos. Table A7 outlines the experimental setups simulated by the model, as in the respective study, with figure numbers given for comparisons of model outputs with the data. The experiments of Helling et al. (2000) and Maboeta et al. (2004) were carried out at 25 °C, those of Zhou et al. (2007) and (2011) at 20 °C. Food quantities were uniformly distributed over the landscape at the feeding times indicated in Table 2. No mortality occurred in the laboratory experiments and so is not considered here.

| **Table A7.** Experimental conditions used in model simulations for comparison with growth and reproduction data. | | | | | |
| --- | --- | --- | --- | --- | --- |
| **Study** | **Our Figure** | **Number of Individuals** | **Food Quantity (g)** | **Feeding Times**  **(days)** | **Observation Period (days)** |
| Helling et al. (2000) | A9 & A10 | 10 | 75, 30, 30, 30 | 0, 35, 42, 49 | 56 |
| Maboeta et al. (2004) | A9 | 20 | 0.54 | 0 | 28 |
| Zhou et al. (2007) | A9 & A10 | 10 | 5 | 0, 28 | 56 |
| Zhou et al. (2011) | A10 | 10 | 5 | 0, 28 | 56 |

*3.1.2. Evaluation of Toxicity Submodels*

Calculations for evaluating the fit of the four potential toxicity submodels (Table A2) to literature data on growth and reproduction are presented in the following section. The model was simulated for each experiment using the different toxicity submodels. In each submodel, the specified parameter values were altered according to the dose-response curves obtained by the data in Fig A3. The value of that parameter under control conditions (0 mg/kg) was the value estimated in Table A1. For example, in toxicity submodel T1 the value of ${IG}_{max}$ at 0 mg/kg is 0.70 g/(day/g) (Table A1), whilst if a chemical is reported to have an effect of e.g. 50% in Fig A3 the value will be altered to 0.35 g(day/g). Model simulation outputs of mean individual biomass (g) and number of cocoons produced of each toxicity submodel was then compared to the experimental data of the respective study to evaluate the goodness of fit. We follow the generally methodology of Anderson (2008) for model selection. Initially, sum of square values for the respective submodel fits to growth and reproduction data were calculated (Table A8).

| **Table A8.** Sum of squares calculated for model outputs of each potential toxicity submodel compared to the literature data in each case study for *G*: growth and *R*: reproduction. | | | | | | | |
| --- | --- | --- | --- | --- | --- | --- | --- |
| **Toxicity Submodel** | **Helling et al. (2000)** | | **Maboeta et al. (2004)** | **Zhou et al. (2007)** | | **Zhou et al. (2011)** | |
|  | ***G*** | ***R*** | ***G*** | ***G*** | ***R*** | ***G*** | ***R*** |
| T1 | 0.30 | 300.4 | 1.067 | 0.174 | 2624 | 0.015 | 600.9 |
| T2 | 0.88 | 139.9 | 1.067 | 0.158 | 36.55 | 0.025 | 374.6 |
| T3 | 0.13 | 38.92 | 1.067 | 0.014 | 3.83 | 0.002 | 2.27 |
| T4 | 0.90 | 228.5 | 0.018 | 0.193 | 623.4 | 0.043 | 376.4 |

In order to account for the difference in scales measured (number of cocoons, individual biomass), sum of square values required normalising, so that each variable is equally weighted by their respective variance. Table A9 presents normalised sum of square values along with variance ratios between growth and reproduction data.

| **Table A9.** Sample size, standard deviations, variances and ratios of variances between growth and reproduction values. | | | | | | | |
| --- | --- | --- | --- | --- | --- | --- | --- |
|  | **Helling et al. (2000)** | | **Maboeta et al. (2004)** | **Zhou et al. (2007)** | | **Zhou et al. (2011)** | |
|  | ***G*** | ***R*** | ***G*** | ***G*** | ***R*** | ***G*** | ***R*** |
| Sample Size | 54 | 5 | 30 | 24 | 6 | 6 | 6 |
| Standard Deviation | 0.185 | 5.90 | 0.068 | 0.041 | 7.31 | 0.05 | 7.77 |
| Variance | 0.034 | 34.80 | 0.0046 | 0.002 | 53.5 | 0.003 | 60.37 |
| Ratio | 1 | 1018 | 0.0046 | 1 | 32227 | 1 | 24582 |

Sum of square values for growth and reproduction data were combined and normalized in each case study by: $\frac{\sum\frac{\hat{\varepsilon}_{i}^{2}}{n}}{Ratio}$ , where $\varepsilon_{i}$ is the normalized difference between the literature data and model output (sum of squares) and *n* is the sample size (Table A9), for growth and reproduction, presented as $\hat{\sigma}^{2}$ values for each toxicity submodel in Table A10.

| **Table A10.** Normalized $\hat{\sigma}^{2}$ values for combined growth and reproduction in each case study compared to the respective toxicity submodels. | | | | |
| --- | --- | --- | --- | --- |
| **Toxicity Submodel** | **Helling et al. (2000)** | **Maboeta et al. (2004)** | **Zhou et al. (2007)** | **Zhou et al. (2011)** |
| T1 | 0.065 | 7.73 | 0.0085 | 0.0033 |
| T2 | 0.044 | 7.73 | 0.0053 | 0.0034 |
| T3 | 0.010 | 7.73 | 0.0005 | 0.0002 |
| T4 | 0.062 | 0.13 | 0.0071 | 0.0046 |

AICc values, expressed as: $AICc=n\log(\hat{\sigma}^{2})+2n'\left( \frac{n}{n-n'-1} \right),$where *n’* is the number of parameters, here represented by the number of toxicity coefficients used in the simulations, are calculated in Table A11 for the $\hat{\sigma}^{2}$ values in Table A9. Numbers of parameters were two in toxicity submodels T2 & T3 and one in the case of T1 & T4. The difference between toxicity submodels (∆*_i_*) was calculated as: $\Delta_{i}= {AICc}_{i}- {AIC}_{min}$ where ${AIC}_{min}$ is the minimum value of *AICc*, to identify the best performing model (Anderson, 2008) (Table A11). Best performing models are represented by values of zero, and increasing values show increasing variance between model outputs and the literature data.

| **Table A11.** AICc and ∆i values for each toxicity submodel and case study, the best performing model represented by a *∆i* value of zero. * indicates the best performing toxicity submodel for a given case study. | | | | | | | | |
| --- | --- | --- | --- | --- | --- | --- | --- | --- |
| **Toxicity Submodel** | **Helling et al. (2000)** | | **Maboeta et al. (2004)** | | **Zhou et al. (2007)** | | **Zhou et al. (2011)** | |
|  | ***AICc*** | ***∆i*** | ***AICc*** | ***∆i*** | ***AICc*** | ***∆i*** | ***AICc*** | ***∆i*** |
| T1 | -159.2 | 108.3 | 63.5 | 122.6 | -140.9 | 82.7 | -66.2 | 30.7 |
| T2 | -180.1 | 87.4 | 65.8 | 124.9 | -152.8 | 70.8 | -62.9 | 34 |
| T3 | -267.5 | 0* | 65.8 | 124.9 | -223.6 | 0* | -96.9 | 0* |
| T4 | -162.0 | 105.5 | -59.1 | 0* | -146.3 | 77.3 | -62.2 | 34.7 |

When adequate food was provided effects of both copper oxychloride and chlorpyrifos on growth and reproduction were best described by supposing physiological parameters $r_{m}$and$r_{B}$ were affected, using toxicity submodel T3. When food was limited, increasing weight loss at higher concentrations was best described by toxicity submodel T4. Table A12 gives evidence ratios (*ER*s) for each toxicity submodel and case study. ER values quantify the level of evidence for supporting an alternative model by comparing the outputs with the best performing model. Here, Table A12 shows that the odds against all toxicity models but the best performing being better are very high, with evidence ratios > 10^6^:1. Higher values provide less support for a model.

| **Table A12.** Evidence ratio (*ER*) values for each toxicity submodel. Values translate how well the models fit the experimental data, with higher *ER* values representing less support. | | | | | |
| --- | --- | --- | --- | --- | --- |
| **Toxicity Submodel** | **Food Availability**  **Sample Size** | **Helling et al. (2000)** | **Maboeta et al. (2004)** | **Zhou et al. (2007)** | **Zhou et al. (2011)** |
|  |  | Optimal | Limited | Near optimal | Near optimal |
|  |  | 59 | 30 | 30 | 12 |
| T1 | ${IG}_{max}$ | 3.3 × 10^23^  9.5 × 10^18^  1  8.1 × 10^22^ | 4.1 × 10^26^  1.3 × 10^27^  1.3 × 10^27^  1 | 9.1 × 10^17^  2.4 × 10^15^  1  6.1 × 10^16^ | 4.6 × 10^6^  2.4 × 10^7^  1  3.4 × 10^7^ |
| T2 | ${IG}_{max} \& r_{m}$ |  |  |  |  |
| T3 | $r_{m} \&$ $r_{B}$ |  |  |  |  |
| T4 | $B_{0}$ |  |  |  |  |

*3.1.3. Model Fits to Sublethal Effects*

Growth and reproduction data for *E. fetida* from experimental studies under various exposures of copper oxychloride and chlorpyrifos are presented here together with the outputs of model simulations run under the same conditions (Table A7). Helling et al. (2000) studied the effect of copper oxychloride on the growth of 10 newly hatched *E. fetida* under optimal feeding conditions. Simulation of the Helling et al. (2000)’s experiment shows good model fits to growth (Fig. A9b) and reproduction (Fig. A10a) data under control and maximum concentrations (Fig. 9b). However, at intermediate concentrations experimental responses do not increase monotonically with concentration and so cannot be fitted by the model. These results are well predicted by submodel T3 in which the allocation of energy to growth and reproduction parameters is directly affected. Effects of copper oxychloride on growth in Maboeta et al. (2004) (Fig. A9c) was not explained by imposing stress on physiological parameters directing the allocation of energy (${IG}_{max}, r_{B}, r_{m}$). As the authors in this case study gave a high density of 20 adult *E. fetida* a limited supply of food at the beginning of their experiment, energy ingestion was restricted, demonstrated by minimal changes in biomass in the control treatment. Rather, the data shows an increase in weight loss with concentration, explained by our energy budget model as the catabolisation of tissue for increasing maintenance requirements. This mechanism is described by submodel T4, resulting in the model outputs presented in Fig. A9d which capture the span of the response. Growth data presented by Zhou et al. (2007) (Fig. A9e) shows great variation in individual biomass between treatment concentrations of chlorpyrifos, with the standard errors for each treatment overlapping. Yet, based on the mean biomasses recorded the model provides a reasonable fit to the growth data (Fig. A9f) and a good fit to the reproduction data (Fig. A10b).

**
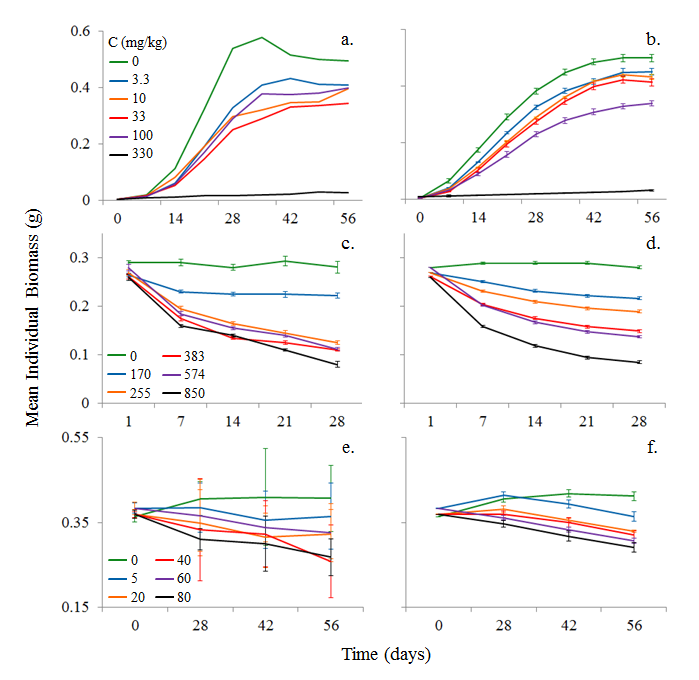
**

**Fig. A9.** Comparison of experimental toxicity data (left-hand panels) and model simulations of toxicity experiments (right hand panels). (a, b) the effects of copper oxychloride (Helling et al. 2000) modelled using submodel T3; c, d) copper oxychloride (Maboeta et al. 2004) using T4; and (e, f) chlorpyrifos (Zhou et al. 2007) using T3.

Zhou et al. (2011) provided the same experimental conditions as Zhou et al. (2007) and recorded mean individual biomass and cocoon production after 56 days exposure as shown in Fig. A10 c & d. Submodel T3 again provides a good fit to the data.


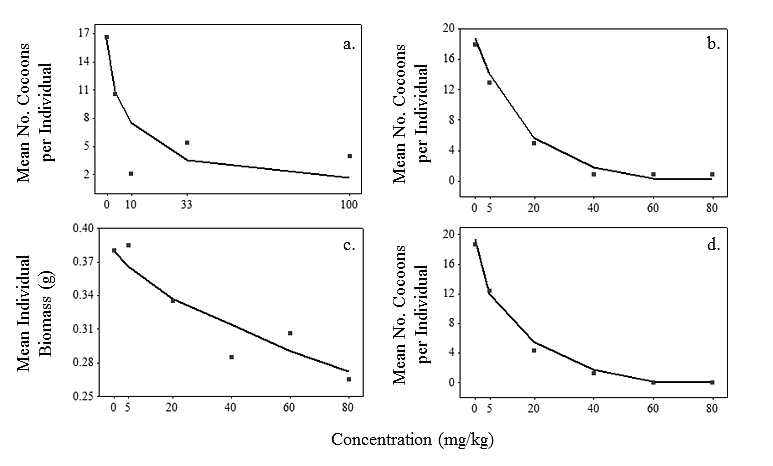


**Fig. A10.** Comparison of model simulations (lines) with toxicity submodel T3 on reproduction (a, b & d) and growth (c) data (points) recorded after 56 days exposure to copper oxychloride (a) and chlorpyrifos (b, c & d) from a) Helling et al. (2000), b) Zhou et al. (2007) and c & d) Zhou et al. (2011),

*3.1.4. Goodness of Fit Tests*

The adequacy of the method of modelling toxicity was evaluated by comparing predictions from the presented model to a ‘random model’. In the ‘random model’ simulations were ran with the parameter values in Table A1 and following the experimental conditions in Table A7, as in the presented model, but with its toxicity coefficient set to a random value. The toxicity coefficient (*k*) in Eq. A6 determines the dose-response curve between concentration and the effect on a physiological parameter. Here we use the best performing toxicity submodel (T3 or T4) (Table A12) and set the value of the toxicity coefficient to random between a narrow range: 0 to twice its measured value. Table A13 shows that predictions of individual growth and reproduction using the regression curves presented in Fig A3 performed better (p < 0.00) than predictions using randomised coefficients in each case. This supports the toxicity dose-response curve fitting methods.

| **Table A13.** Results of randomisation tests evaluating the adequacy of the toxicity submodel method. In each randomisation the toxicity coefficient (*k*) was chosen randomly from a uniform distribution ranging from zero to twice its value. Goodness of fit was calculated as the sum of squared differences between model predictions and the data shown in Figs A9 – A13. *N* = number of randomisations; *p* = proportion of random coefficient models that fit the data better (have less sum of squares) than the presented coefficients used to represent dose-response relationships in Eq. 5. | | | |
| --- | --- | --- | --- |
| **Study** | **Fig.** | ***N*** | ***p*** |
| Helling et al. (2000) | A9b. | 50 | 0.00 |
| Maboeta et al. (2004) | A9d. | 50 | 0.00 |
| Zhou et al. (2007) | A9f. | 50 | 0.00 |
| Helling et al. (2000) | A10a. | 50 | 0.00 |
| Zhou et al. (2007) | A10b. | 50 | 0.00 |
| Zhou et al. (2011) | A10c. | 50 | 0.00 |
| Zhou et al. (2011) | A10d. | 50 | 0.00 |

Fig. A11 presents histograms of the sums of squares in the randomisation tests presented in Table A13. The sum of squares for the presented model is represented by the arrow on the x-axis of each graph. Higher sums of squares indicate worse fits between model outputs and data. The p values in Table A13 record the frequency with which randomised-parameter models fitted better than the presented model. It is evident from Fig A11 that the presented model provided a substantially better fit to the data in each case.


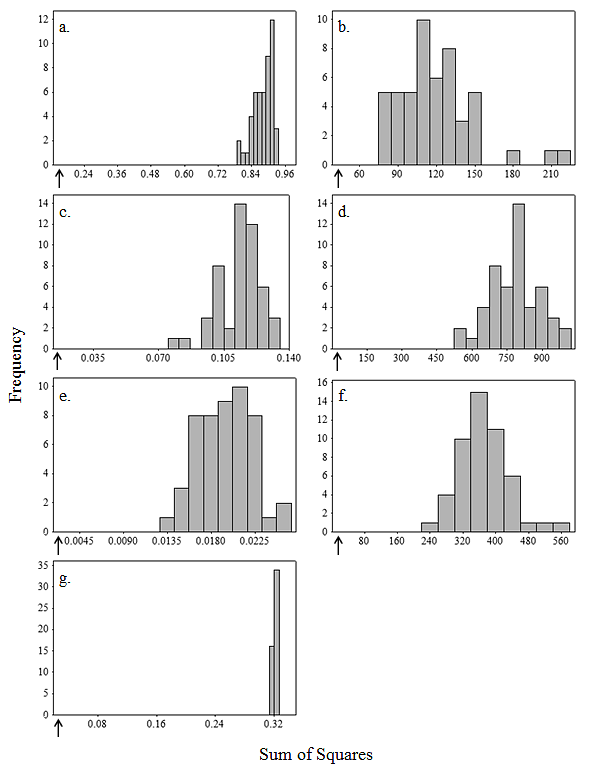


**Fig. A11.** Histograms showing the goodness of fit of the presented model (arrow) and randomized-toxicity coefficient models for a, c, e & g: growth and b, d & f: reproduction for a & b: Helling et al. (2000); c & d: Zhou et al. (2007); e & f: Zhou et al. (2011) and g: Maboeta et al. (2004). See text for further details.

*3.2. Uncertainty Analysis*

Taking a POM approach to validation of the results, good model fits to the data show a high degree of certainty in model outputs. However, in some cases the literature data contained significant noise, making it difficult to interpret the goodness of fit of the model. For instance, growth data presented by Zhou et al. (2007) (Fig. A9e) shows great variation in individual biomass between treatment concentrations, with the standard errors for each treatment overlapping. Yet, the model is less variable, with standard errors usually much lower than in the simulated experimental study. Model simulations may thus be more reliable than experimental studies, but only if we have confidence in the parameters on which the model is based. Rigorous testing of the toxicity submodel approach in Section *3.1.2* and goodness of fits tests between the data and model outputs in Section *3.1.4* support the model as being robust.

As few of the parameters of the model were available from the literature, calculations were necessary to translate available data to energetic units in a transparent way (Section *1.4.*). The sensitivity analysis shows these values to be rather robust (Table A4), with parameters causing the most variation in model outputs being *E* and $E_{c}$. Errors in these parameters would affect temperature- dependent and production-related metabolic rates respectively. Yet, these parameters were taken directly from the literature and fits to the data suggest their estimates are reliable. The greatest threat to the model would be uncertainties surrounding *IG_max_* and the different mass thresholds *M_p_* and *M_m_*, as these parameters manage the availability of energy for expenditure to life cycle processes, either directly through governing individual energy intake or indirectly by altering the mass-dependence of metabolic rates. However, the model fits to independent data suggest that these values are realistic as all simulations depend on these parameters, particularly under depleting food conditions. In general, it is difficult to reach conclusions as to the uncertainty of the model as energetic parameter values are rarely known with certainty.

*3.3. Recommendations*

Good model fits to the toxic effects of copper oxychloride and chlorpyrifos on sublethal endpoints growth and reproduction highlights the importance of accounting for energy budgets. Identifying the toxicity submodel best explaining the observed effects allows interpretation of lower tier toxicity results, for extrapolation to the population level. The physiological pathway of copper oxychloride under food limitation was different to that under adequate food supplies. This finding provides an ecologically relevant interpretation, and extrapolations to the field population level, where food limitation commonly occurs, should combine toxicity submodels T3 and T4. In conclusion, the synthesis of this information provides a robust foundation for ecological inferences, and suggests how extrapolations may be made between laboratory toxicity studies and field population dynamics.

**References**

Dickschen, F. & Topp, W. (1987) Feeding activities and assimilation efficiencies of *Lumbricus rubellus* (Lumbricidae) on a plant-only diet. *Pedobiologia*, **30**, 31–37.

SANCO (2010) *SANCO 11803/2010 Working document amending Commission Regulation (EU) No xxxx/2010 laying down the requirements for the dossier to be submitted for the authorisation of plant protection products.* European Commission.
