# Supplementary material for: An energy budget agent-based model of earthworm populations and its application to study the effects of pesticides
Source: Ecol Modell. 2014 May 24;280:5–17. doi: 10.1016/j.ecolmodel.2013.09.012 (PMC4375675; doi:10.1016/j.ecolmodel.2013.09.012)
Supplement: Supplementary file 2 [file mmc2.docx]

**Appendix B: Experimental Data and Model Outputs**

*Figure 4 – Individual growth and reproduction*

| **Table B1.** Comparison of mean individual biomass (g) measures recorded by Gunadi et al. (2002) and Gunadi & Edwards (2003) with model outputs, as presented in Fig 4a & 4e of the manuscript respectively. | | | | | | | | |
| --- | --- | --- | --- | --- | --- | --- | --- | --- |
| **Gunadi et al. (2002)** | | | **Gunadi and Edwards (2003)** | | | | | |
| **Time (days)** | **Data** | **Model** | **Time (days)** | **Data** | **Model** | **Time (days)** | **Data** | **Model** |
| 7 | 0.011 | 0.011 | 0 | 0.011 | 0.011 | 217 | 0.360 | 0.353 |
| 14 | 0.032 | 0.040 | 7 | 0.024 | 0.048 | 224 | 0.372 | 0.350 |
| 21 | 0.079 | 0.106 | 14 | 0.060 | 0.116 | 231 | 0.392 | 0.350 |
| 28 | 0.131 | 0.183 | 21 | 0.094 | 0.193 | 238 | 0.361 | 0.346 |
| 35 | 0.256 | 0.256 | 28 | 0.170 | 0.261 | 245 | 0.390 | 0.341 |
| 42 | 0.368 | 0.317 | 35 | 0.249 | 0.296 | 252 | 0.390 | 0.339 |
| 49 | 0.414 | 0.366 | 42 | 0.308 | 0.333 | 259 | 0.387 | 0.345 |
| 56 | 0.442 | 0.404 | 49 | 0.358 | 0.357 | 266 | 0.386 | 0.348 |
| 63 | 0.462 | 0.431 | 56 | 0.370 | 0.376 | 273 | 0.390 | 0.350 |
| 70 | 0.448 | 0.451 | 63 | 0.376 | 0.388 | 280 | 0.394 | 0.354 |
| 77 | 0.450 | 0.466 | 70 | 0.374 | 0.392 | 287 | 0.390 | 0.352 |
| 84 | 0.459 | 0.476 | 77 | 0.372 | 0.391 | 294 | 0.376 | 0.346 |
| 91 | 0.494 | 0.483 | 84 | 0.396 | 0.396 | 301 | 0.365 | 0.340 |
| 98 | 0.495 | 0.488 | 91 | 0.390 | 0.400 | 308 | 0.360 | 0.336 |
| 105 | 0.490 | 0.492 | 98 | 0.378 | 0.402 | 315 | 0.358 | 0.335 |
| 112 | 0.482 | 0.494 | 105 | 0.367 | 0.404 | 322 | 0.468 | 0.359 |
| 119 | 0.447 | 0.496 | 112 | 0.348 | 0.403 | 329 | 0.532 | 0.387 |
| 126 | 0.439 | 0.492 | 119 | 0.335 | 0.402 | 336 | 0.502 | 0.402 |
| 133 | 0.422 | 0.473 | 126 | 0.340 | 0.396 | 343 | 0.489 | 0.410 |
| 140 | 0.412 | 0.455 | 133 | 0.341 | 0.400 | 350 | 0.470 | 0.410 |
| 147 | 0.400 | 0.437 | 140 | 0.342 | 0.399 | 357 | 0.440 | 0.410 |
| 154 | 0.402 | 0.420 | 147 | 0.335 | 0.391 | 364 | 0.458 | 0.411 |
| 161 | 0.387 | 0.404 | 154 | 0.339 | 0.379 | 371 | 0.382 | 0.410 |
| 168 | 0.402 | 0.388 | 161 | 0.315 | 0.366 | 378 | 0.378 | 0.407 |
| 175 | 0.393 | 0.372 | 168 | 0.340 | 0.359 | 385 | 0.382 | 0.404 |
| 182 | 0.341 | 0.357 | 175 | 0.475 | 0.374 | 392 | 0.384 | 0.403 |
| 189 | 0.333 | 0.342 | 182 | 0.480 | 0.376 | 399 | 0.382 | 0.403 |
|  |  |  | 189 | 0.425 | 0.372 | 406 | 0.378 | 0.398 |
|  |  |  | 196 | 0.395 | 0.367 | 413 | 0.370 | 0.392 |
|  |  |  | 203 | 0.362 | 0.361 | 420 | 0.360 | 0.387 |
|  |  |  | 210 | 0.358 | 0.358 |  |  |  |

| **Table B2.** Comparison of mean individual biomass (g) and cocoon production measures recorded by Reinecke & Viljoen (1990) with model outputs, as presented in Fig 4e, 4b & 4d of the manuscript respectively. | | | | | | |
| --- | --- | --- | --- | --- | --- | --- |
| **Time (days)** | **Fig 4e: Mean Individual Biomass (g)** | | **Fig 4b: Number of Cocoons per 10 days** | | **Fig 4d: Number of Cocoons per 10 days** | |
|  | Data | Model | Data | Model | Data | Model |
| 0 | 0.32 | 0.31 | 0 | 0 | 0 | 0 |
| 10 | 0.35 | 0.34 | 23 | 30 | 8 | 14 |
| 20 | 0.36 | 0.35 | 31 | 31 | 34 | 32 |
| 30 | 0.30 | 0.33 | 40 | 39 | 23 | 26 |
| 40 | 0.30 | 0.31 | 38 | 36 | 10 | 12 |
| 50 | 0.27 | 0.28 | 44 | 41 | 0 | 2 |
| 60 | 0.16 | 0.26 | 46 | 36 | 1 | 0 |
| 70 | 0.47 | 0.34 | 52 | 48 | 30 | 28 |
| 80 | 0.44 | 0.41 | 43 | 40 | 46 | 49 |
| 90 | 0.44 | 0.38 | 42 | 44 | 32 | 34 |
| 100 | 0.41 | 0.36 | 41.5 | 42 | 12 | 16 |
| 110 | 0.32 | 0.33 | 44 | 38 | 2 | 4 |
| 120 | 0.30 | 0.31 | 36 | 36 | 0 | 0 |
| 130 | 0.14 | 0.29 | 50 | 42 | 0 | 0 |
| 140 | 0.05 | 0.26 | 36 | 41 | 0 | 0 |
| 150 | 0.35 | 0.34 | 32 | 34 | 0 | 18 |
| 160 | 0.66 | 0.34 | 36 | 40 | 24 | 26 |
| 170 |  |  | 33 | 36 | 54 | 24 |
| 180 |  |  | 34 | 40 | 30 | 16 |

*Figure 5 – Copper Oxychloride and Chlorpyrifos Effects on Individual Biomass*

| **Table B3.** Comparison of mean individual biomass (g) measures at different concentrations of copper oxychloride recorded by Helling et al. (2000) with model outputs, as presented in Fig 5a & 5b of the manuscript respectively. | | | | | | |
| --- | --- | --- | --- | --- | --- | --- |
| **Time (days)** | **0 mg/kg** | | **3.3 mg/kg** | | **10 mg/kg** | |
|  | Data | Model | Data | Model | Data | Model |
| 0 | 0.003 | 0.004 | 0.004 | 0.003 | 0.003 | 0.004 |
| 7 | 0.020 | 0.065 | 0.015 | 0.041 | 0.018 | 0.037 |
| 14 | 0.112 | 0.175 | 0.061 | 0.131 | 0.083 | 0.113 |
| 21 | 0.325 | 0.291 | 0.192 | 0.235 | 0.191 | 0.205 |
| 28 | 0.540 | 0.383 | 0.330 | 0.325 | 0.298 | 0.289 |
| 35 | 0.580 | 0.450 | 0.409 | 0.383 | 0.322 | 0.362 |
| 42 | 0.515 | 0.486 | 0.433 | 0.416 | 0.346 | 0.416 |
| 49 | 0.500 | 0.500 | 0.411 | 0.449 | 0.350 | 0.441 |
| 56 | 0.495 | 0.500 | 0.409 | 0.451 | 0.396 | 0.434 |
| **Time (days)** | **33 mg/kg** | | **100 mg/kg** | | **330 mg/kg** | |
|  | Data | Model | Data | Model | Data | Model |
| 0 | 0.003 | 0.005 | 0.003 | 0.003 | 0.004 | 0.009 |
| 7 | 0.016 | 0.028 | 0.015 | 0.033 | 0.009 | 0.012 |
| 14 | 0.054 | 0.104 | 0.059 | 0.089 | 0.013 | 0.014 |
| 21 | 0.148 | 0.195 | 0.170 | 0.158 | 0.016 | 0.016 |
| 28 | 0.252 | 0.274 | 0.289 | 0.230 | 0.018 | 0.019 |
| 35 | 0.289 | 0.347 | 0.378 | 0.279 | 0.020 | 0.022 |
| 42 | 0.331 | 0.399 | 0.376 | 0.309 | 0.023 | 0.025 |
| 49 | 0.336 | 0.423 | 0.380 | 0.328 | 0.029 | 0.028 |
| 56 | 0.344 | 0.414 | 0.399 | 0.340 | 0.028 | 0.031 |

| **Table B4.** Comparison of mean individual biomass (g) measures at different concentrations of copper oxychloride recorded by Maboeta et al. (2004) with model outputs, as presented in Fig 5c & 5d of the manuscript respectively. | | | | | | |
| --- | --- | --- | --- | --- | --- | --- |
| **Time (days)** | **0 mg/kg** | | **170 mg/kg** | | **255 mg/kg** | |
|  | Data | Model | Data | Model | Data | Model |
| 1 | 0.290 | 0.280 | 0.265 | 0.270 | 0.270 | 0.270 |
| 7 | 0.291 | 0.289 | 0.230 | 0.251 | 0.195 | 0.231 |
| 14 | 0.280 | 0.289 | 0.225 | 0.232 | 0.165 | 0.210 |
| 21 | 0.293 | 0.289 | 0.225 | 0.222 | 0.145 | 0.196 |
| 28 | 0.281 | 0.280 | 0.222 | 0.216 | 0.125 | 0.189 |
| **Time (days)** | **383 mg/kg** | | **574 mg/kg** | | **850 mg/kg** | |
|  | Data | Model | Data | Model | Data | Model |
| 1 | 0.262 | 0.262 | 0.280 | 0.280 | 0.260 | 0.260 |
| 7 | 0.175 | 0.204 | 0.185 | 0.202 | 0.160 | 0.159 |
| 14 | 0.135 | 0.176 | 0.155 | 0.168 | 0.140 | 0.118 |
| 21 | 0.125 | 0.158 | 0.140 | 0.147 | 0.110 | 0.095 |
| 28 | 0.110 | 0.149 | 0.112 | 0.137 | 0.080 | 0.085 |

| **Table B5.** Comparison of mean individual biomass (g) measures at different concentrations of chlorpyrifos recorded by Zhou et al. (2007) with model outputs as presented in Fig 5e & 5f of the manuscript respectively. | | | | | | |
| --- | --- | --- | --- | --- | --- | --- |
| **Time (days)** | **0 mg/kg** | | **5 mg/kg** | | **20 mg/kg** | |
|  | Data | Model | Data | Model | Data | Model |
| 0 | 0.364 | 0.364 | 0.384 | 0.384 | 0.370 | 0.370 |
| 28 | 0.406 | 0.406 | 0.385 | 0.414 | 0.349 | 0.382 |
| 42 | 0.410 | 0.419 | 0.356 | 0.394 | 0.316 | 0.355 |
| 56 | 0.407 | 0.412 | 0.365 | 0.364 | 0.323 | 0.330 |
| **Time (days)** | **40 mg/kg** | | **60 mg/kg** | | **80 mg/kg** | |
|  | Data | Model | Data | Model | Data | Model |
| 0 | 0.369 | 0.369 | 0.383 | 0.383 | 0.369 | 0.369 |
| 28 | 0.333 | 0.369 | 0.366 | 0.361 | 0.310 | 0.346 |
| 42 | 0.323 | 0.350 | 0.338 | 0.333 | 0.300 | 0.318 |
| 56 | 0.258 | 0.321 | 0.326 | 0.306 | 0.268 | 0.291 |

*Figure 6 – Copper Oxychloride & Chlorpyrifos Effects on Reproduction and Chlorpyrifos Effects on Final Biomass*

| **Table B6.** Comparison of cocoon production measures after 56 days exposure to different concentrations of copper oxychloride recorded by Helling et al. (2000) and chlorpyrifos recorded by Zhou et al. (2007) with model outputs, as presented in Fig 6a & 6b of the manuscript respectively. | | | | | |
| --- | --- | --- | --- | --- | --- |
| **Helling et al. (2000)** | | | **Zhou et al. (2007)** | | |
| Concentration (mg/kg) | Data | Model | Concentration (mg/kg) | Data | Model |
| 0 | 16.66 | 16.4 | 0 | 18 | 18.8 |
| 3.3 | 10.63 | 10.9 | 5 | 13 | 14.1 |
| 10 | 2.1 | 7.6 | 20 | 5 | 5.7 |
| 33 | 5.4 | 3.6 | 40 | 1 | 1.8 |
| 100 | 4 | 1.7 | 60 | 1 | 0.4 |
|  |  |  | 80 | 1 | 0.3 |

| **Table B7.** Comparison of mean individual biomass (g) and cocoon production measures after 56 days exposure to different concentrations chlorpyrifos recorded by Zhou et al. (2011) with model outputs, as presented in Fig 6c & 6d of the manuscript respectively. | | | | |
| --- | --- | --- | --- | --- |
|  | **Fig 6c: Mean Individual Biomass (g)** | | **Fig 6d: Mean Number of Cocoons per Individual** | |
| Concentration (mg/kg) | Data | Model | Data | Model |
| 0 | 0.380 | 0.380 | 18.75 | 19.4 |
| 5 | 0.385 | 0.366 | 12.5 | 12 |
| 20 | 0.335 | 0.337 | 4.38 | 5.5 |
| 40 | 0.285 | 0.314 | 1.25 | 1.8 |
| 60 | 0.306 | 0.290 | 0 | 0.2 |
| 80 | 0.265 | 0.272 | 0 | 0 |

*Figure 7 – Field Population Density & Biomass*

| **Table B8.** Comparison of field population density and biomass measures recorded by Monroy et al. (2006) with model outputs, as presented in Figs 7a – 7g of the manuscript. | | | | | | | | |
| --- | --- | --- | --- | --- | --- | --- | --- | --- |
|  | **Mean Population Density (Individuals per m^2^)** | | | | | | | |
|  | **Total** | | **Adult** | | **Juvenile** | | **Cocoon** | |
| Season | Data | Model | Data | Model | Data | Model | Data | Model |
| Autumn | 2450 | 3053 | 1680 | 1060 | 690 | 1345 | 0 | 648 |
| Winter | 3965 | 3903 | 1760 | 1618 | 1790 | 1320 | 1260 | 965 |
| Spring | 8460 | 7096 | 3810 | 2634 | 3070 | 2210 | 3970 | 2252 |
| Summer | 4888 | 4366 | 432 | 848 | 3992 | 2516 | 184 | 1002 |
|  | **Mean Population Biomass (kg per m^2^)** | | | | | |  |  |
|  | **Total** | | **Adult** | | **Juvenile** | |  |  |
| Season | Data | Model | Data | Model | Data | Model |  |  |
| Autumn | 0.78 | 0.61 | 0.55 | 0.34 | 0.15 | 0.26 |  |  |
| Winter | 0.98 | 0.92 | 0.70 | 0.64 | 0.26 | 0.28 |  |  |
| Spring | 1.57 | 1.21 | 1.22 | 0.86 | 0.37 | 0.34 |  |  |
| Summer | 0.53 | 0.56 | 0.13 | 0.27 | 0.39 | 0.31 |  |  |
